# A Subregion of Insular Cortex Is Required for Rapid Taste-Visceral Integration and Consequent Conditioned Taste Aversion and Avoidance Expression in Rats

**DOI:** 10.1523/ENEURO.0527-21.2022

**Published:** 2022-07-06

**Authors:** A-Hyun Jung, Camille Tessitore King, Ginger D. Blonde, Michael King, Camilla Griggs, Koji Hashimoto, Alan C. Spector, Lindsey A. Schier

**Affiliations:** 1 Neuroscience Graduate Program, University of Southern California, Los Angeles, CA 90089; 2 Department of Psychology, Stetson University, DeLand, FL 32723; 3 Department of Psychology and Program in Neuroscience, Florida State University, Tallahassee, FL 32306; 4 Department of Biology, Stetson University, DeLand, FL 32723; 5 Department of Biological Sciences, University of Southern California, Los Angeles, CA 90089; 6 Department of Morphological and Physiological Sciences, Kumamoto University, Kumamoto, Japan 860-8555

**Keywords:** brain mapping, food reward, gustatory cortex, interoception, taste hedonics, visceral cortex

## Abstract

Postingestive signals are important for shaping appetitive and consummatory responses, but the brain mechanisms required to assimilate interoceptive events with those at the frontlines of ingestion (taste-guided) are poorly understood. Here, we investigated whether an insular cortex (IC) region, which receives viscerosensory input, including gustatory, is required to modify taste-elicited consummatory reactions in response to a real-time interoceptive change using a serial taste reactivity (TR) test where the rats’ oromotor and somatic reactions to intraoral (IO) infusions of sucrose were periodically assessed over 45 min following lithium chloride (LiCl) administration. Results showed that neurally-intact rats shifted from an ingestive repertoire to an aversive one as LiCl took effect. Overall, this hedonic shift was delayed in rats with bilateral neurotoxic IC lesions. Rats with greater neuronal loss in posterior gustatory IC displayed fewer aversive reactions to sucrose following this initial LiCl injection. We further assessed whether the failure to integrate interoceptive feedback with ongoing taste-guided behavior impaired acquisition and/or expression of conditioned aversion and/or avoidance in these same rats. Although, as a group, LiCl-injected rats with IC lesions subsequently avoided the sugar in a 48-h two-bottle test, their preference for sucrose was significantly greater than that of the LiCl-injected neurally-intact rats. Overall lesion size, as well as proportion of the posterior gustatory and/or anterior visceral IC were each associated with impaired avoidance. These findings reveal new roles for the posterior gustatory and anterior visceral ICs in multisensory integrative function.

## Significance Statement

Adaptive eating and drinking behaviors require that the brain incorporates interoceptive state information into appropriate taste-guided appetitive and consummatory responses in real time and over the long-term through learning. Here, we show for the first time that loss of function in a subregion of insular cortex (IC) that receives both general visceral and gustatory sensory inputs hinders rapid adjustments to consummatory behaviors in response to a negative interoceptive event. We further show that this primary integrative deficit precludes robust adaptive avoidance behavior that normally safeguards against enduring the same visceral consequences again. Collectively, the findings yield new insights into the neural organization of taste-interoceptive integration in IC, which subserves key aspects of short-term and long-term control of ingestive behavior.

## Introduction

Survival depends in part on the ability to rapidly detect, integrate, and respond to sensory events associated with eating and drinking. The insular cortex (IC), which receives both exteroceptive and interoceptive inputs and is highly interconnected with motivational effector circuits, has long been implicated in these types of convergent processes that enable adaptive ingestive decisions (Krushel and van der Kooy, 1988; Allen et al., 1991; Fontanini and Katz, 2006, 2009; Parkes et al., 2015; Gogolla, 2017; Gehrlach et al., 2020; Vincis et al., 2020; Boughter and Fletcher, 2021). Conditioned taste avoidance (CTAvoid) is the most well-studied example of this phenomenon. In CTAvoid, the orosensory properties of an ingested substance (e.g., flavor; conditioned stimulus; CS) become associated with its negative interoceptive consequences (e.g., nausea; unconditioned stimulus; US) such that future encounters with that flavor recall the negative event and prevent its ingestion again (Garcia et al., 1955). Over the last 40 years, numerous studies have shown that the gustatory IC (GC) is necessary to successfully express a CTAvoid (Yamamoto et al., 1980; Braun et al., 1982; Lasiter and Glanzman, 1982; Dunn and Everitt, 1988; Bermudez-Rattoni and McGaugh, 1991; Rosenblum et al., 1995, 1997; Naor and Dudai, 1996; Nerad et al., 1996; Schafe and Bernstein, 1998; Cubero et al., 1999; Fresquet et al., 2004; Roman and Reilly, 2007) and recent studies have further attributed this to a specific IC subregion comprising the contiguous posterior GC and anterior visceral cortex (pGC/aVC; Schier et al., 2014, 2016).

**Figure 1.**
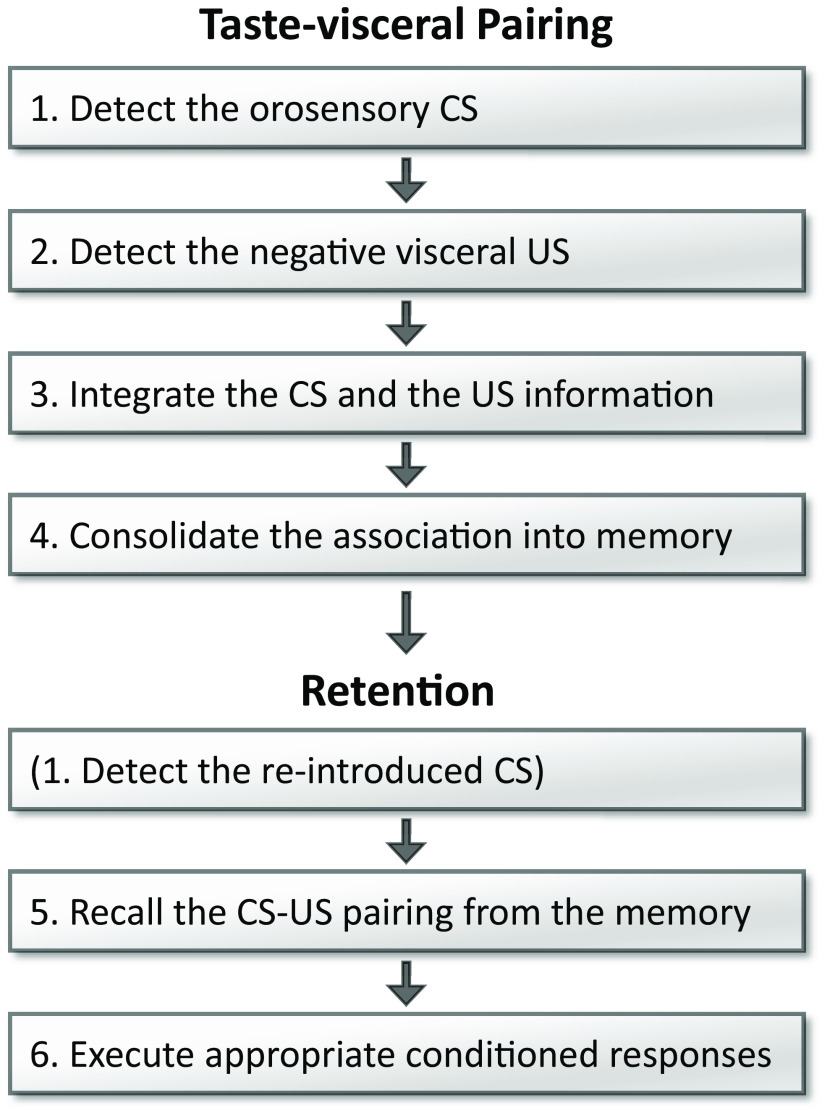
Principal processes to establish and express CTAvoid and/or CTAver. To successfully learn CTAvoid/aver, an animal must detect the orosensory CS, detect the negative visceral event, assimilate those sensory events, and consolidate the association into memory. Then, to later express the learning when re-introduced to a CS, the subject must detect the CS, recall the previous association from memory, and perform the conditioned response. Failure to accomplish any of these pairing and retention steps, would impair the ultimate expression of the learned avoidance/aversion. CS, conditioned stimulus; US, unconditioned stimulus.

However, CTAvoid, like many ingestive phenomena, is mechanistically complex. In order to successfully reject a flavor CS, the brain must (1) detect the orosensory CS; (2) detect the negative US; (3) associatively link the CS and US; (4) consolidate the association into memory; (5) recall it from memory when the CS is reintroduced; and (6) execute an appropriate response (Spector et al., 1988; Reilly and Bornovalova, 2005; Schier and Spector, 2019; Fig. 1). Disruption in any of these functionally distinct processes would lead to a similar outcome: failure to avoid ingesting the solution at test. Thus, one aim of the present study was to clarify which of these CTAvoid-related events the pGC/aVC is involved in. Based on a prior study, this IC subregion is not necessary to detect the taste CS and interoceptive US (steps 1–2; Schier et al., 2016), but whether it is required to integrate and consolidate these inputs (steps 3–4) and whether that relates to subsequent deficits in CTAvoid expression (steps 5–6) are unknown.

Moreover, in addition to CTAvoid, USs can foster a fundamentally different type of learned response to the CS: conditioned taste aversion (CTAver; Pelchat et al., 1983; Parker, 2003; Schier et al., 2019). Whereas taste avoidance prevents the anticipated negative outcome, taste aversion refers to the negative hedonic evaluation of the taste stimulus, which itself limits ingestion. The difference in these processes is evident in how animals respond to different types of visceral events associated with food. For example, whereas food poisoning will alter a food’s palatability such that its flavor is rendered aversive (i.e., CTAver), lactose malabsorption will cause animals to avoid consuming lactose-containing substances, but without affecting its hedonic appeal (i.e., CTAvoid; Pelchat et al., 1983; Parker, 2003; Schier et al., 2019). Failure to avert the CS ingestion in preference and intake tests does not reveal which of these motivational processes is affected by a given experimental manipulation. But, rodents, like humans, display distinct stereotypic oromotor and somatic responses to positively-valenced and negatively-valenced taste stimuli (Grill and Norgren, 1978) and measurement of these responses in a taste reactivity (TR) test provides a readout on the hedonic appeal of a taste stimulus, in the absence of goal-directed motives like avoidance.

**Figure 2.**
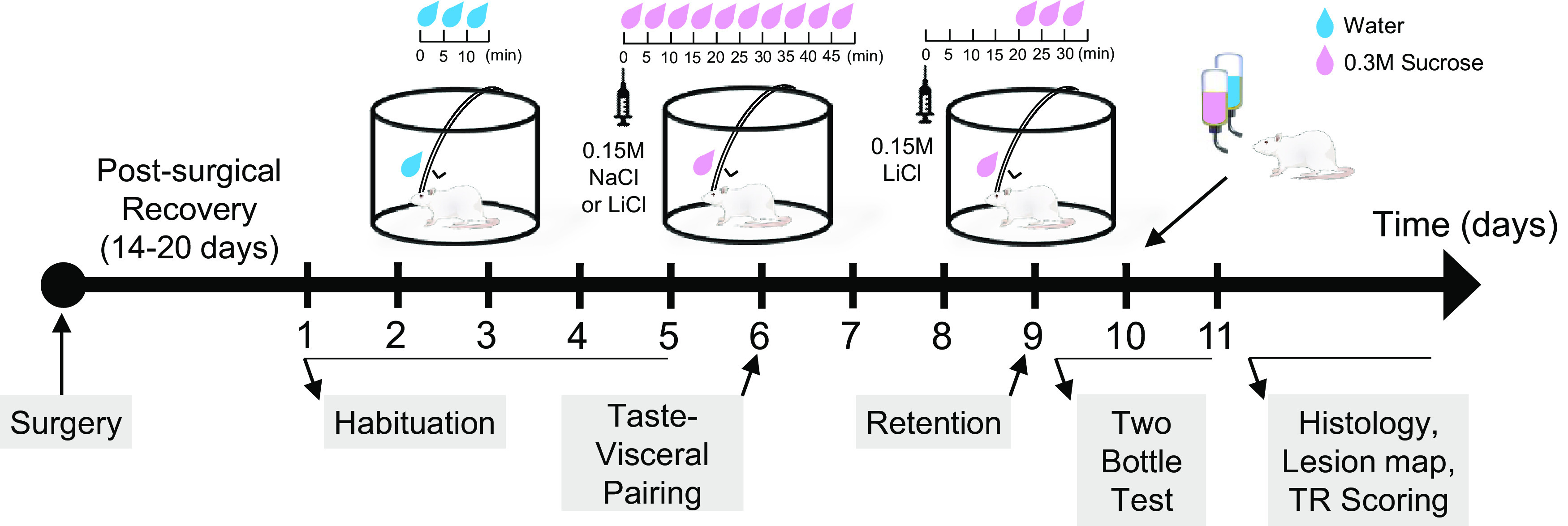
The experimental design and timeline. The experimental timeline showing when (in days) each of the behavioral tests was conducted beginning with habituation, labeled below the black horizontal arrow. The injection and infusion schedule within each session is shown above the diagram. TR tests were conducted in a TR chamber and two-bottle tests were performed in the home cage. Following the completion of two-bottle test, histologic analyses including lesion mapping were completed and TR videos were scored in a blinded fashion. NaCl, Sodium Chloride; LiCl, Lithium Chloride; TR, taste reactivity, M, Molar, Min, Minute.

To determine whether the pGC/aVC, which is necessary for CTAvoid, is also required to adaptively shift the hedonic value of the taste CS in response to the emetic event (CTAver), we used the serial TR paradigm (Spector et al., 1988). In the serial TR pairing session, lithium chloride (LiCl) is injected to induce nausea and then a novel tastant, sucrose, is infused into the oral cavity while oromotor and somatic reactions are recorded. As the LiCl takes effect, intact rats rapidly shift TR from an ingestive repertoire to an aversive one. Therefore, here, we assessed whether loss of function in pGC/aVC disrupted the online integration of interoceptive cues with incoming taste information while minimizing the memory requirement. Next, because the coincident presentation of the taste CS and the LiCl US in this initial pairing session is sufficient to condition an aversion to the CS in intact rats (Spector et al., 1988), we tested whether loss of function in pGC/aVC results in subsequent CTAver and/or CTAvoid deficits (Fig. 2).

## Materials and Methods

### Subjects

Adult male Sprague Dawley rats, weighing 330–420 g at the start of the experiment, were singly housed in polycarbonate shoebox cages with *ad libitum* access to rodent chow [Teklad Rodent Diet (Envigo, Indianapolis) for phase 1, Purina 5001 for phase 2] and deionized water. The climate-controlled colony room was set to 12/12 h light/dark cycle. The experiment was run in two phases (*n* = 48 for phase 1, n = 30 for phase 2). Phase 1 was conducted at Stetson University and phase 2 was conducted at the University of Southern California. All behavioral procedures and schedules were identical between the phases. The experiment was performed in successive replications during each phase. All surgical and behavioral procedures were approved by the Stetson University (phase 1) and the University of Southern California (phase 2) Animal Care and Use Committees.

### Surgery

#### Stereotaxic lesions

After at least 5 d of acclimation to the colony room, rats were subdivided into four surgical groups (IC_2_ lesion, IC_3_ lesion, IC_2_+IC_3_ lesion, or Sham-operated control, see below; Table 1). Lesions were targeted to the IC_2_ region as defined by Schier et al. (2014), which spans 0.2–1.2 mm anterior to bregma and includes the pGC and overlying anterior VC, and the IC_3_ region, which spans the VC from −0.8 to +0.2 mm from bregma, or both regions (IC_2_+IC_3_). The number of animals and corresponding stereotaxic coordinates used for each group and phase are listed in Table 1. All the lesions were systematically mapped postmortem by an experimenter blinded to the experimental treatments and only the animals that met the lesion criteria were included for the final analyses (see below). Stereotaxic surgeries were performed under sterile conditions with anesthesia [ketamine hydrochloride (125 mg/kg) and xylazine (5 mg/kg) mixture for phase 1; isoflurane (5% induction rate, ∼2.5–3% maintenance rate) for phase 2]. After the rat was completely anesthetized, the incision area on the scalp was shaved and cleaned with 70% ethanol and betadine. The rat’s head was secured in a stereotaxic apparatus with blunt ear bars. An incision was made in the skin to expose the skull. The skull was leveled against bregma and lambda by adjusting the bite bar. Small holes were made in the skull above the target coordinates on each side using a micro hand drill. A 1-μl Hamilton syringe filled with either phosphate buffered saline (0.1 m PBS, for sham surgeries) or ibotenic acid (IBO; 20 mg/ml in 0.1 m PBS, for lesion surgeries) was connected to a glass micropipette tip (outside diameter ∼50 μm) with a paraffin wax seal. Using bregma as a reference point, the glass micropipette was positioned at the target coordinate (Table 1). PBS or IBO was slowly infused in two or three microinfusions (volumes listed in Table 1), separated by an ∼2-min interval to allow diffusion into the tissue. Infusions were done bilaterally. After all injections were administered, cranial holes were filled with hemostatic sponge.

**Table 1:** Lesion groups, training groups, and stereotaxic coordinates in each phase

			Stereotaxic target coordinates+							
			IC_2_				IC_3_			
Phase/*N*s	Lesion Group	CTA Group/*N*s	AP	ML	DV	Vol. (μl)	AP	ML	DV	Vol. (μl)
Phase 1 *N* = 29	Sham IC_2_+IC_3_ (PBS)	NaCl (*N* = 5)	+0.6	±5.7	−6.6, −6.7	0.18	−0.5	± 6.2	−7.0	0.18
		LiCl (*N* = 6)								
	Bilateral IC_2_ (IBO)	NaCl (*N* = 0)	+0.6	±5.7	−6.6, −6.7	0.18	N/A			
		LiCl (*N* = 3)								
	Bilateral IC_3_ (IBO)	NaCl (*N* = 2)	N/A				−0.5	± 6.2	−7.0	0.18
		LiCl (*N* = 3)								
	Bilateral IC_2_+IC_3_ (IBO)	NaCl (*N* = 3)	+0.5	± 5.9	−6.6, −6.7	0.18	−0.5	± 6.2	−7.0	0.18
		LiCl (*N* = 7)								
Phase 2 *N* = 18	Sham IC_2_+IC_3_ (PBS)	NaCl (*N* = 4)	+0.6	±5.8	−6.7	0.1	−0.5	± 5.9	−7.0	0.1
		LiCl (*N* = 5)								
	Bilateral IC_2_ (IBO)	NaCl (*N* = 0)	+0.6	±5.8	−6.7	0.1	N/A			
		LiCl (*N* = 3)								
	Bilateral IC_3_ (IBO)	NaCl (*N* = 0)	N/A				−0.5	± 5.9	−7.0	0.1
		LiCl (*N* = 0)								
	Bilateral IC_2_+IC_3_ (IBO)	NaCl (*N* = 0)	+0.6	±5.8	−6.7	0.1	−0.5	± 5.9	−7.0	0.1
		LiCl (*N* = 6)								

Sample sizes (*N*) include animals that underwent surgery, met the lesion criteria and completed all behavioral tests. ^+^ All stereotaxic coordinates were based on the Paxinos and Watson stereotaxic atlas (6th Edition). CTA, conditioned taste aversion; IC, insular cortex; NaCl, sodium chloride; LiCl, lithium chloride; AP, anterior-posterior; ML, medial-lateral; DV, dorsal-ventral; Vol, volume; PBS, phosphate buffered saline; IBO, 20 mg/ml ibotenic acid in PBS; LiCl, Lithium chloride; NaCl, Sodium chloride.

#### Intraoral (IO) cannulation

Immediately following the stereotaxic surgery, the anesthetized rat was placed in a supine position for bilateral IO cannula implantation. IO cannulation was performed according to the procedures adapted from (Phillips and Norgren, 1970; Grill and Norgren, 1978). Briefly, polyethylene tubing (PE 100) was inserted just anterolateral to the second maxillary molar and tunneled under the facial muscles to an exit site at the top of the head. The tubes were outfitted with a metal connector pin and anchored to the skull with microscrews and dental cement. After the cement head cap was fully cured, the skin was closed around it with single stay sutures. During surgery and for 3 d after surgery, rats were given analgesic (Carprofen, 5 mg/kg, sc) and antibiotic (Enrofloxacin, 2.3 mg/kg, sc). Powdered chow and/or wet chow mash were provided during the 14–20 d of recovery.

### Behavioral procedures

#### TR: habituation, CTA acquisition, and retention

The TR chamber comprised a cylindrical chamber with a transparent Plexiglas floor. The infusion tubing ran from a 10-ml syringe in an external infusion pump (Harvard Apparatus) through a single channel stainless steel swivel mounted at the top of the TR chamber. The free end of the tubing was encased in a spring tether and connected to the rat’s IO cannula at the connector pin. This system allowed the rat to move freely around the chamber, while preventing it from accessing the infusion line tubing. A camera was mounted on a tripod aimed at an angled mirror below the chamber (phase 1) or was mounted directly below the chamber (phase 2). After recovery from the surgery, rats were habituated to the TR chamber across 5 d (10 min/d). On the first habituation day, the rat was simply placed in the chamber. On the second day, the rat was connected to the tubing, but no infusions were made. On days 3–5, the rat was connected to the tubing and received infusions of deionized water (1 ml/min) for 30 s every 5 min (min 0, 5, and 10). At the end of each 10-min session, the rat was returned to the home cage.

Rats from each surgical group were further subdivided into treatment groups for the taste-visceral pairing session (Table 1). On day 6, rats received an intraperitoneal injection of either 0.15 m LiCl (13.33 ml/kg) to induce visceral malaise or isomolar NaCl (13.33 ml/kg) as a control directly before the start of the session. Then the rat was placed in the TR chamber and its IO cannula was connected to the infusion line. Sucrose (0.3 m) was IO infused at a rate of 1 ml/min for 30 s (from the first oromotor response) every 5 min, beginning at minute 0, for a total of 10 infusions. After the last infusion at minute 45, the rat was disconnected from the tubing and returned to its home cage. The rats’ oromotor and somatic responses to sucrose during each infusion period were video recorded and analyzed later offline (see below).

Three days later, rats were given a TR retention test to determine whether the sucrose-LiCl pairing yielded a CTAver. In this test, all rats were intraperitoneally injected with 0.15 m LiCl (13.33 ml/kg), placed in the chamber and connected to the infusion line (min 0). Starting at minute 20, 0.3 m sucrose was infused for 30 s at a rate of 1 ml/min every 5 min up to minute 30 (total of three infusions). After the last infusion, the rat was returned to its home cage. The rats’ oromotor and somatic responses to sucrose during each infusion period were video recorded and analyzed later offline (see below). The LiCl was injected during this test because it has been shown that to observe the full display of the conditioned response, the visceral state during conditioning and testing must be concordant (Spector et al., 1988).

#### Two-bottle preference test (24, 48 h)

After the TR retention test, two bottles were placed on the home cage to measure CTAvoid. One bottle was filled with deionized water and the other one was filled with 0.3 m sucrose. After 24 h, the amount of each solution that the rat consumed was recorded (by weight, to the nearest 0.1 g). The bottles were refilled with fresh sucrose solution and deionized water and the position of two bottles on the home cage was switched. After another 24 h, the amount consumed from each bottle was recorded.

### Behavioral analyses

#### TR

Before scoring, videos recorded during the serial TR taste-visceral pairing and retention sessions were zoomed and cropped to include the rats’ face and forelimbs using Sony Movie Studios software. A subset of videos spanning the initial taste-visceral pairing session (min 5, 15, 25, and 35) and all three videos from the TR retention session were viewed in slow motion (frame by frame) while individual ingestive and aversive oromotor and somatic responses were counted by an experimenter blinded to the experimental history of the subjects. These responses were categorized into four stereotypic ingestive TR behaviors and four stereotypic aversive TR behaviors, as described previously (Spector et al., 1988) and outlined in Extended Data Figure 4-4. Ingestive TR behaviors included: tongue protrusion, lateral tongue protrusion, mouth movement, and paw lick. Aversive TR behaviors included: gape, chin rub, head shake, and forelimb flail. Raw counts were used for each of these behaviors, except paw lick. The duration of paw licking was recorded in seconds and then multiplied by 6 to get an estimated count. Passive drips were recorded but not included in the final scores. The counts of individual ingestive behaviors were summated for a total ingestive TR score. Likewise, the counts of individual aversive behaviors were summated for a total aversive TR score. In some cases, the face or paws were obscured; these were categorized as no data and the duration was noted. For videos that had >1 s of no data, individual behavior categories of the ingestive and aversive TR responses were summed and corrected separately for lost time by dividing each score by the scorable time and then multiplying that by 30 which is the duration of a single infusion. Then, the counts of individual ingestive behaviors and aversive behaviors were summated, respectively, to derive a total ingestive and aversive TR score.

10.1523/ENEURO.0527-21.2022.f4-4 Extended Data Figure 4-4 Definitions of oromotor and somatic reactivity behaviors. Corresponds to Figures 4 and 5. Download Figure 4-4, DOC file.

#### Two-bottle preference

Based on the intake records (in grams), sucrose preference over the first 24 h and entire 48-h period was calculated by dividing sucrose intake over the total solution intake (sucrose + water) and multiplying by 100.

### Histology and lesion mapping

#### Histology

The rats were euthanized by overdose with an intraperitoneally injected euthanasia agent containing sodium pentobarbital at the end of the experiment. Transcardial perfusion was performed with saline followed by 4% paraformaldehyde in 0.1 m PBS (PFA). Then, the skull was placed on a stereotaxic apparatus and leveled to match dorsoventral coordinates at bregma and lambda by adjusting the bite bar. Using a hand drill, a band of skull parallel to but slightly anterior to lambda was removed to expose the brain. The brain was then blocked by moving a blade mounted on a stereotaxic holder from left to right. This blocking method was done to ensure that each brain was sectioned in the correct stereotaxic plane. The remaining skull pieces were manually removed and the brain was stored in 4% PFA at 4°C until sectioning. The brains were sliced with vibratome (Leica, VT1200) in serial 50-μm coronal sections and mounted on gelatin-coated slides. The slides were dried, Nissl stained with thionin and coverslipped to visualize cytoarchitecture and lesion in and around the IC.

#### Lesion mapping

**Figure 3.**
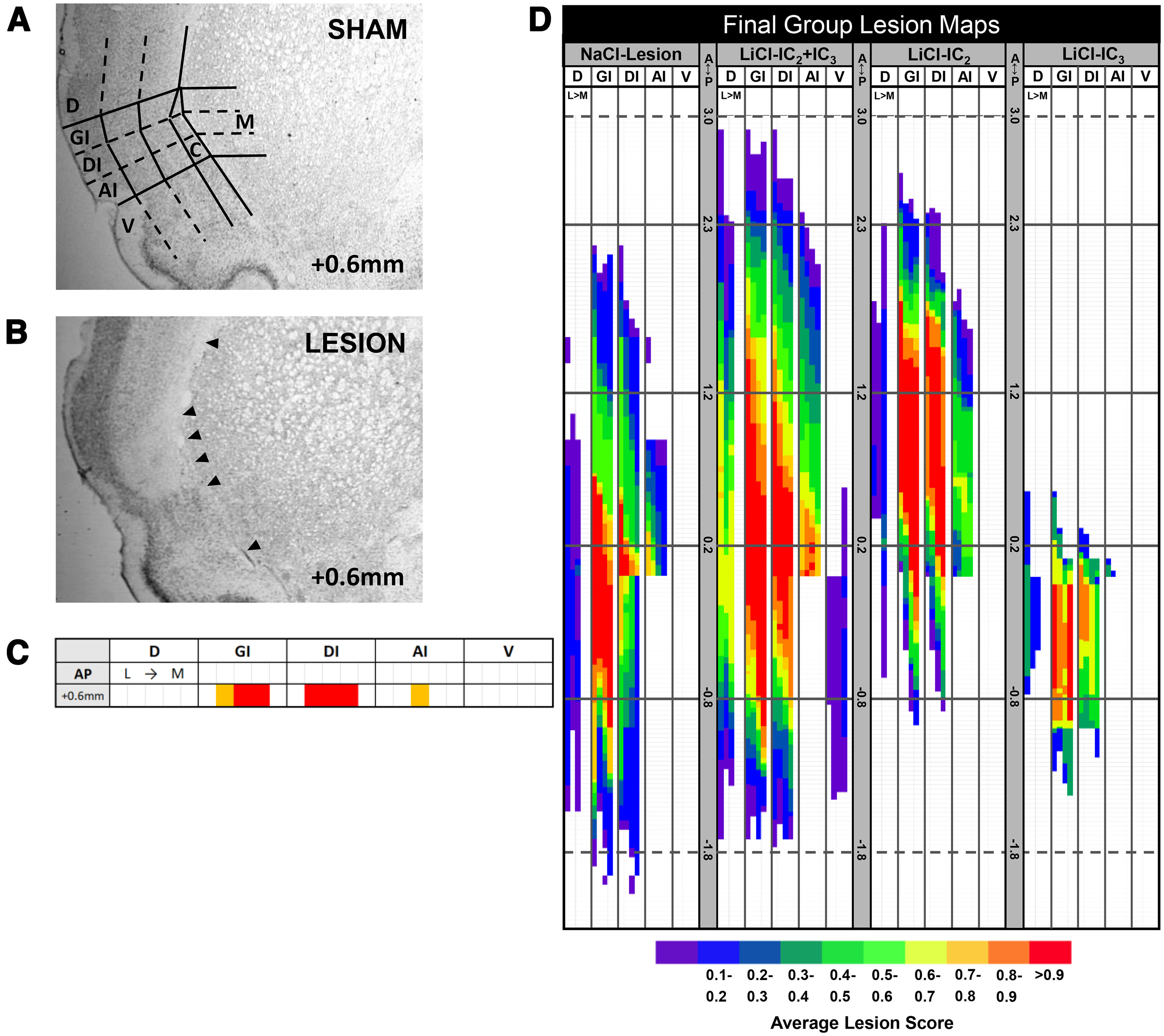
Representative photomicrograph of brain sections with delineated grids of IC and surrounding region and group-wise overlap lesion maps in a color-coded manner on 2D grids. A sham (***A***) and a lesion brain section (***B***) at AP coordinate of +0.6 mm are shown. Black arrowheads indicate the approximate borders of each layer in ***B***. An example of the corresponding color-coded lesion map for ***B*** is shown in panel ***C***. The overlap maps (***D***) show the average lesion scores in color scale for the NaCl-lesion (including NaCl-IC_3_ and NaCl-IC_2_+IC_3_), LiCl-IC_2_, LiCl-IC_3_, and LiCl-IC_2_+IC_3_ groups. Solid and dotted lines indicate different AP levels relative to bregma including IC_2_ borders (+1.2 and +0.2 mm) and IC_3_ borders (+0.2 and −0.8 mm). Representative lesion maps and corresponding photomicrographs of brain sections of IC_2_, IC_3_, and IC_2_+IC_3_ lesion are shown in Extended Data Figures 3-1, 3-2, and 3-3, respectively. A, anterior to bregma; P, posterior to bregma; D, dorsal to the granular layer (GI); GI, granular IC; DI, dysgranular IC; AI, agranular IC (dorsal to the rhinal fissure); V, ventral to rhinal fissure; C, claustrum (medial to GI, DI, and AI); M, medial to claustrum; LiCl, Lithium chloride; NaCl, Sodium chloride; IC, Insular cortex.

The lesion mapping system developed by Schier et al. (2016) enables a thorough and quantitative reconstruction of lesion size and location in 50-μm brain sections onto 2D Microsoft Excel grid. The IC in each coronal section is delineated into three layers (GI: granular, DI: dysgranular, AI: agranular, dorsal to the rhinal fissure anterior to AP 0.0 mm) and each layer is equally divided into three columnal grids along the mediolateral axis. The same segmentation is repeated for the areas dorsal to IC (D) and ventral to the rhinal fissure (V). Note that AI is included in V at AP levels posterior to 0.0 mm. For D and V, the area mapped is equivalent to the dorsoventral distance occupied by the GI, DI, and AI layers in IC in that same section. The left and right hemisphere are separately mapped. Brain sections were examined under 1.25× magnification to assess the extent of neuronal loss. In each grid cell for each brain section, the extent of cellular loss was scored on a trinary scale as complete (entire region devoid of cells; red), partial (at least half of the region, but less than the entire region, devoid of cells; yellow) or none (less than half of the region devoid of cells; white) and color-coded onto the 2D Excel grid. On the Excel grid, each row represents a single 50-μm coronal section from anterior to posterior. Each column is a subdivision of IC (GI, DI, AI) and surrounding area (D, V) which is further divided into five medial-to-lateral subcolumns. These five subcolumns comprise the medial to lateral thirds of insular cortical tissue (three columns), claustrum which is medial to IC (1 column), and the area medial to the claustrum (1 column). Representative images of the delineated grids on brain sections and 2D plotting on excel grid are shown in Figure 3*A–C*. Lesions were mapped and scored by an experimenter blind to the subjects’ surgery and treatment conditions. Fixation and histology cause some shrinkage in the brain tissue. Therefore, to ensure that the lesion maps were in register with the stereotaxic atlas and with one another (Paxinos and Watson, 2007), a landmark correction procedure was applied as follows. The AP level of coronal sections at key histologic landmarks were recorded on the Excel spreadsheet. These landmarks included the anterior start of the striatum, the joining of the corpus callosum, the disappearance of the indusium grisium below the corpus callosum, the rostral joining of the anterior commissure, the emergence of the CA3 field in hippocampus, among others. Once these landmarks were noted, the number of sections between one landmark and the next landmark was counted and divided into the actual stereotaxic difference represented in the atlas. This gave a correction factor for each 50-μm section between the two particular landmarks. In essence, the relative AP distance that a single 50-μm section represented in the corresponding row on the mapping grid was determined by multiplying the section thickness (50 μm) by the local correction factor. This also allowed us to get a better estimate of the AP level relative to the two flanking landmarks. Once the entire lesion in both hemispheres was mapped separately and the AP scale was corrected, a third map (symmetry) was generated by comparing the lesion scores of each grid cell from two hemispheres and assigning the lower one to the corresponding grid cell in the symmetry map. Total lesion score was calculated by adding the total value of all grid cells in each rat’s symmetry map, regardless of where the lesion was located. Region of interest lesion scores (e.g., IC_2_ or IC_3_) were calculated as the total value of all grid cells in the designated region divided by the total number of cells in that region on the symmetry map to get a proportion of the area with lesion. For IC_2_ and IC_3_, this included grid cell values in AI, DI, and GI, above the rhinal fissure.

10.1523/ENEURO.0527-21.2022.f3-1 Extended Data Figure 3-1 Representative IC2+IC3 lesion map and corresponding photomicrographs of brain sections in both hemispheres. IC2+IC3 lesion brain sections at the coordinates of +1.2 mm (top), +0.2 mm (middle), –0.8 mm (bottom) in the left and right hemispheres (right and left column, respectively) with black arrowheads indicating the borders of each layer in IC and surrounding region. Middle column shows representative IC2+IC3 lesion maps of each hemisphere with a symmetry lesion map in the middle. Solid and dotted lines on the map indicate different AP levels relative to bregma including IC2 borders (+1.2 and +0.2 mm) and IC3 borders (+0.2 and –0.8 mm). A, anterior to bregma, P, posterior to bregma, D, dorsal to the granular layer (GI); GI, granular IC; DI, dysgranular IC; AI, agranular IC (dorsal to the rhinal fissure); V, ventral to rhinal fissure. Download Figure 3-1, TIF file.

10.1523/ENEURO.0527-21.2022.f3-2 Extended Data Figure 3-2 Representative IC2 lesion map and corresponding photomicrographs of brain sections in both hemispheres. IC2 lesion brain sections at the coordinates of +1.2 mm (top), +0.2 mm (middle), –0.8 mm (bottom) in the left and right hemispheres (right and left column, respectively) with black arrowheads indicating the borders of each layer in IC and surrounding region. Middle column shows representative IC2 lesion maps of each hemisphere with a symmetry lesion map in the middle. Solid and dotted lines on the map indicate different AP levels relative to bregma including IC2 borders (+1.2 and +0.2 mm) and IC3 borders (+0.2 and –0.8 mm). A, anterior to bregma, P, posterior to bregma, D, dorsal to the granular layer (GI); GI, granular IC; DI, dysgranular IC; AI, agranular IC (dorsal to the rhinal fissure); V, ventral to rhinal fissure. Download Figure 3-2, TIF file.

10.1523/ENEURO.0527-21.2022.f3-3 Extended Data Figure 3-3 Representative IC3 lesion map and corresponding photomicrographs of brain sections in both hemispheres. IC3 lesion brain sections at the coordinates of +1.2 mm (top), +0.2 mm (middle), –0.8 mm (bottom) in the left and right hemispheres (right and left column, respectively) with black arrowheads indicating the borders of each layer in IC and surrounding region. Middle column shows representative IC3 lesion maps of each hemisphere with a symmetry lesion map in the middle. Solid and dotted lines on the map indicate different AP levels relative to bregma including IC2 borders (+1.2 and +0.2 mm) and IC3 borders (+0.2 and –0.8 mm). A, anterior to bregma, P, posterior to bregma, D, dorsal to the granular layer (GI); GI, granular IC; DI, dysgranular IC; AI, agranular IC (dorsal to the rhinal fissure); V, ventral to rhinal fissure. Download Figure 3-3, TIF file.

Finally, to compare lesion size and placement across individual rats or groups of rats, all rats’ symmetry lesion maps were extended and put on the same AP scale. To accomplish this, the AP value for each row was rounded to the nearest multiple of 10. Then, each of those rows was subdivided into 10-μm rows. For example, if the corrected AP distance of a row was designated as 81 μm, then it was rounded to 80 μm and further subdivided into eight 10-μm rows. To generate group-wise lesion maps across rats meeting a specified criterion (see below), individual symmetry maps were aligned along these 10-μm-scaled AP coordinates. The proportion of rats in that group with lesion in each grid cell was calculated and converted to color scale to visualize areas of lesion overlap among rats in each group. Final group lesion maps are shown in Figure 3*D*.

### Data analyses

Percentage of the subregions of interest (i.e., IC_2_, IC_3_) with lesion were calculated for each rat as described above. Only rats with ≥50% bilateral lesion in IC_2_ and IC_3_ were included in the IC_2_+IC_3_ group. Rats with ≥50% bilateral lesion in IC_2_, but <50% cell loss in IC_3_ were included in the IC_2_ group. Rats with ≥50% bilateral lesion in IC_3_, but <50% lesion in IC_2_ were included in the IC_3_ group. Based on these lesion criteria, rats were divided into three groups for the main statistical analyses— NaCl-injected control, LiCl-injected Sham, LiCl-injected IC_2_+IC_3_ lesion. Because there were no differences among NaCl-injected rats with Sham or IC_2_ and/or IC_3_ lesion, they were combined into one group (data shown for comparison purposes in Extended Data Fig. 4-1). Any rat whose lesion score did not meet these criteria was excluded from statistical analyses. Because of low sample sizes, rats that met the IC_2_ or IC_3_ lesion criteria were not included in these main group-wise analyses but see Extended Data Figures 4-2 and 4-3. Because we observed non-normal distributions in some of the behavioral data, nonparametric Mann–Whitney tests were used to determine group-wise statistical differences. Wilcoxon signed rank tests were used for within-group paired comparisons. The p-values were corrected for multiple comparison with the Benjamini–Hochberg false discovery correction procedure (Benjamini and Hochberg, 1995). In order to examine patterns of behavior across the multiple behavioral assessments, each individual rat’s behavior performance was converted to a robust Z-score standardized against the median and deviation of the LiCl Sham group’s performance on the respective behavioral measurements. Spearman’s rank correlations were used to determine whether overall lesion size (including IC, D, V, claustrum and the region medial to claustrum) and/or lesion in specific parts of IC were related to behavioral performance (robust Z-score). All LiCl-injected rats with lesion in IC_2_, IC_3_, and IC_2_+IC_3_ were included in these correlational tests and no correction procedure for multiple comparison was applied.

**Figure 4.**
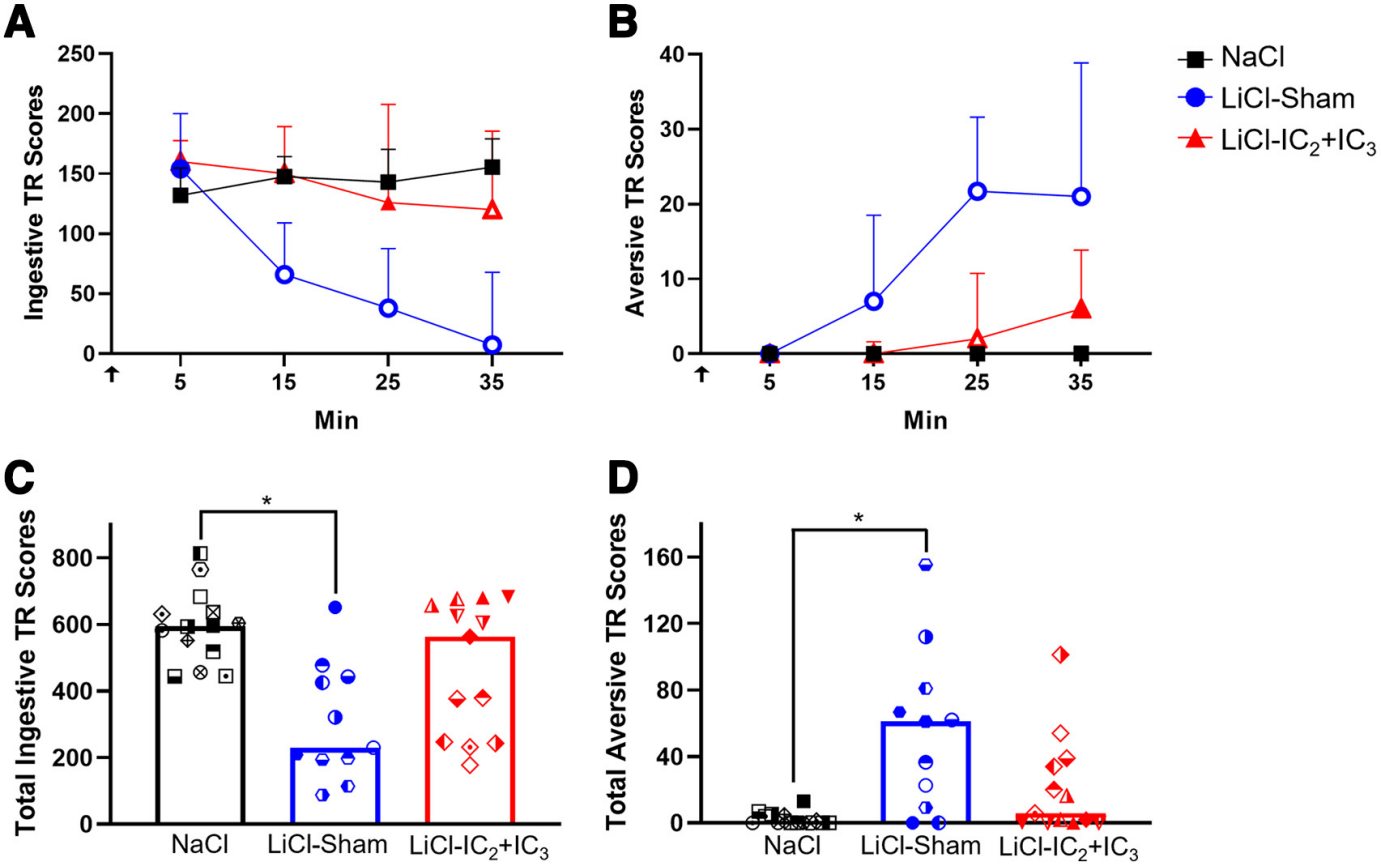
Lesions in IC_2_ and IC_3_ disrupted the adaptation of oromotor taste responses to sucrose following intraperitoneal LiCl injections on the taste-visceral pairing session. Categories and definitions of oromotor and somatic reactivity behaviors are shown in Extended Data Figure 4-4. Because there were no differences among NaCl-injected rats with sham or IC_2_ and/or IC_3_ lesion, they were combined into one group (data shown in Extended Data Figs. 4-1 and 4-5). ***A***, ***B***, Median (+ Semi-IQR) values of ingestive (***A***) and aversive (***B***) TR scores induced by 30-s IO sucrose infusions across the initial taste-visceral pairing session, plotted as a function of time following the intraperitoneal injection of either NaCI (*n* = 14) or LiCI (*n* = 11 for Sham; *n* = 13 for IC_2_+IC_3_ lesion). Black arrow indicates the approximate time of the intraperitoneal injection. Open symbols represent time points that the respective LiCl-injected groups were statistically different from the NaCl group (*p* ≤ 0.05). ***C***, ***D***, Median values of total ingestive (***C***) and aversive (***D***) TR scores during taste-visceral pairing session plotted with different symbols for individual animals in each group. Rats are assigned to the same symbols in each figure. Statistical significances are adjusted based on Benjamini–Hochberg false discovery rate for multiple comparisons and indicated as asterisks. Statistics are in Tables 3-5. LiCl-injected rats with IC_2_ or IC_3_ lesion were not included because of low sample sizes but their behavioral performances are in Extended Data Figures 4-2 and 4-3. LiCl, Lithium chloride; NaCl, Sodium chloride; TR, Taste reactivity.

10.1523/ENEURO.0527-21.2022.f4-1 Extended Data Figure 4-1 Lesions in IC2 and/or IC3 did not affect the behavior performance in NaCl-injected rats. A, Median (+ Semi-IQR) ingestive (left) and aversive (right) TR scores to IO sucrose infusions in acquisition session following NaCl injection (black arrow) in sham (n = 9) or lesion group (n = 5). B, Median total ingestive (left) and aversive (right) TR scores during acquisition are plotted with the data points indicating individual animals. C, Median (+ Semi-IQR) ingestive (left) and aversive (right) TR scores in retention session as a function of time following the LiCl injection (black arrow) in sham (n = 8) or lesion group (n = 5). D, Median total ingestive (left) and aversive (right) TR scores during retention are plotted with individual data points. E, Median sucrose preference over water (in percentage) during the first 24 h (left) or 48 h (right) in two-bottle test are plotted with individual data points. A–E, Histograms with the same letter were not statistically different (all ps > 0.05). Statistics are in Extended Data Figure 4-5. Download Figure 4-1, TIF file.

10.1523/ENEURO.0527-21.2022.f4-2 Extended Data Figure 4-2 A, Median (+ Semi-IQR) ingestive (left) and aversive (right) TR scores across the taste-visceral pairing session following the intraperitoneal injection of either NaCI or LiCI (black arrow) are plotted. B, C, Median value of total ingestive (left) and aversive (right) TR scores during pairing (B) and retention (C) are plotted with different symbols for individual animals in each group. D, Median sucrose preferences over water in percentage during the first 24 h (left) or 48 h (right) of two-bottle test are plotted with individual data points. NaCl group (n = 13–14), LiCl-sham group (n = 11), LiCl-IC2 group (n = 5–6), and LiCl- IC2+IC3 group (n = 12–13). Download Figure 4-2, TIF file.

10.1523/ENEURO.0527-21.2022.f4-3 Extended Data Figure 4-3 A, Median (+ Semi-IQR) ingestive (left) and aversive (right) TR scores across the taste-visceral pairing session following the intraperitoneal injection of either NaCI or LiCI (black arrow) are plotted. B, C, Median value of total ingestive (left) and aversive (right) TR scores during pairing (B) and retention (C) are plotted with different symbols for individual animals in each group. D, Median sucrose preferences over water in percentage during the first 24 h (left) or 48 h (right) of two-bottle test are plotted with individual datapoints. NaCl group (n = 13–14), LiCl-sham group (n = 11), LiCl-IC3 group (n = 3), and LiCl- IC2+IC3 group (n = 12–13). Download Figure 4-3, TIF file.

## Results

### Lesion and groups

Only rats that had at least 50% bilateral lesion of IC_2_ and IC_3_ were included in the main group-wise analyses and compared with both the histologically verified Sham controls injected with LiCl and the group injected with NaCl during the initial taste-visceral pairing session. Group sample sizes are included in Table 1. Group-wise lesion topographic maps are shown in Figure 3*D*. Representative IC_2_+IC_3_, IC_2_, IC_3_ lesion maps and photomicrographs are found in Extended Data Figures 3-1, 3-2, 3-3. Behavioral data from rats with ≥50% bilateral lesion in IC_2_ or IC_3_ are shown in Extended Data Figures 4-2 and 4-3 for comparison but were not included in the overall group-wise analyses because of the limited sample sizes.

### Behavior

#### Extensive lesions in IC_2_+IC_3_ disrupted taste-guided oromotor and somatic adaptive responding to LiCl-induced malaise

First, we asked whether rats with extensive IC_2_+IC_3_ lesions capably alter TR and somatic behaviors elicited to the CS (IO sucrose infusions) as LiCl takes effect in an initial CS-US pairing session. At 5 min after the initial intraperitoneal injection of LiCl or NaCl, all groups displayed comparably high levels of ingestive TR, with virtually no aversive responses (Fig. 4*A*,*B*). Whereas the NaCl-injected group sustained high levels of ingestive responding and low levels of aversive responding across the entire CS-US pairing session, the LiCl-injected Sham group reduced its ingestive responses and increased its aversive responses at minutes 25 and 15, respectively, compared with the minute 5 baseline levels. On the other hand, the LiCl-injected IC lesion group was slower to diverge from baseline (Fig. 4*A*,*B*; Table 2). Group-wise analyses further showed that by minute 15 and throughout the remainder of the session LiCl-injected Sham rats significantly reduced their ingestive responses and increased aversive responses to sucrose relative to the NaCl-injected group. The LiCl-injected IC lesion group failed to alter their ingestive and aversive response patterns at the key inflection points observed for the LiCl-injected Sham group (minutes 25 and 15, respectively; Tables 3, 4). The LiCl-injected IC lesion group suppressed its ingestive responses and increased its aversive responses later in the session (at minutes 35 and 25, respectively) relative to the NaCl-injected group (Fig. 4*A*,*B*; Tables 3, 4). When scores were summed across all four IO infusion time points, the LiCl-injected Sham group displayed significantly fewer ingestive and more aversive responses than the NaCl-injected group (Fig. 4*C*,*D*; Table 5). The LiCl IC_2_+IC_3_ group exhibited an intermediate level of cumulative ingestive and aversive TR, which were not different from the NaCl group or the LiCl Sham group (Fig. 4*C*,*D*; Table 5). Because there was considerable behavioral variability within each group on this CS-US pairing session (see Fig. 4*C*,*D*), Spearman’s rank-order correlations were conducted to determine whether total lesion size or lesion size within IC subregions of interest were associated with a failure to adapt TR in response to LiCl injections. These analyses showed that more extensive neuronal loss in IC_2_+IC_3_, and more specifically within the posterior half of IC_2_, was linked to a more tempered display of aversive reactivity (Table 6). Extended Data Figures 4-2*A* and 4–3*A* show the comparative performance of small samples of LiCl-injected rats with lesions localized to either IC_2_ or IC_3_ on this taste-visceral pairing session.

**Table 2:** Ingestive or aversive TR scores at successive time points versus minute 5 baseline for each group on the taste-visceral pairing session

Ingestive scores			
	Na	Sham-Li (q* = 0.033)	IC_2_+IC_3_-Li (q* = 0.0167)
Minute 5 vs 15	0.54	0.083	0.34
Minute 5 vs 25	0.178	0.024+	0.110
Minute 5 vs 35	0.178	0.024+	0.002+
Aversive scores			
	Na	Sham-Li (q* = 0.05)	IC_2_+IC_3_-Li (q* = 0.033)
Minute 5 vs 15	0.313	0.008+	0.313
Minute 5 vs 25	0.313	0.004+	0.016+
Minute 5 vs 35	0.56	0.004+	0.016+

Corresponds to Figure 4*A,B*. Wilcoxon signed-rank tests were conducted and the significance levels within each group were adjusted based on Benjamini–Hochberg false discovery rate for multiple comparisons (q*; Benjamini and Hochberg, 1995). Values with a plus symbol (+) are statistically significant after correction. IC, Insular cortex; Li, Lithium chloride; Na, Sodium chloride.

**Table 3:** Comparison of ingestive TR scores between groups at select time points across the taste-visceral pairing session

Minute 5, n.s.			
	Na	Sham-Li	IC_2_+IC_3_-Li
Na		0.8825	0.4802
Sham-Li			0.2828
Minute 15, q* = 0.0333			
	Na	Sham-Li	IC_2_+IC_3_-Li
Na		0.0011+	0.7093
Sham-Li			0.0104+
Minute 25, q* = 0.0167			
	Na	Sham-Li	IC_2_+IC_3_-Li
Na		<0.0001+	0.2555
Sham-Li			0.1021
Minute 35, q* = 0.0333			
	Na	Sham-Li	IC_2_+IC_3_-Li
Na		0.0004+	0.008+
Sham-Li			0.4498

Corresponds to Figure 4*A*. Significance level was adjusted based on Benjamini–Hochberg false discovery rate for multiple comparisons (q*; Benjamini and Hochberg, 1995). Values with a plus symbol (+) are statistically significant after correction. n.s. = none significant. IC, Insular cortex; Li, Lithium chloride; Na, Sodium chloride.

**Table 4:** Comparison of aversive TR scores between groups at select time points across the taste-visceral pairing session

Minute 5, n.s.			
	Na	Sham-Li	IC_2_+IC_3_-Li
Na		0.1748	0.3259
Sham-Li			>0.9999
Minute 15, q* = 0.0167			
	Na	Sham-Li	IC_2_+IC_3_-Li
Na		0.0021+	0.3845
Sham-Li			0.0446
Minute 25, q* = 0.05			
	Na	Sham-Li	IC_2_+IC_3_-Li
Na		<0.0001+	0.0146+
Sham-Li			0.0385+
Minute 35, q* = 0.0167			
	Na	Sham-Li	IC_2_+IC_3_-Li
Na		0.0007+	0.0501
Sham-Li			0.0689

Corresponds with Figure 4*B*. Significance level was adjusted based on Benjamini–Hochberg false discovery rate for multiple comparisons (q*; Benjamini and Hochberg, 1995). Values with a plus symbol (+) are statistically significant after correction. n.s. = none significant. IC, Insular cortex; Li, Lithium chloride; Na, Sodium chloride.

**Table 5:** Comparison of total ingestive or aversive TR score between groups for the taste-visceral pairing session

Total ingestive score, q* = 0.0167			
	Na	Sham-Li	IC_2_+IC_3_-Li
Na		0.0002+	0.2555
Sham-Li			0.0350
Total aversive score, q* = 0.0167			
	Na	Sham-Li	IC_2_+IC_3_-Li
Na		0.001+	0.0479
Sham-Li			0.0590

Corresponds to Figure 4*C,D*. Significance level was adjusted based on Benjamini–Hochberg false discovery rate for multiple comparisons (q*; Benjamini and Hochberg, 1995). Values with a plus symbol (+) are statistically significant after correction. IC, Insular cortex; Li, Lithium chloride; Na, Sodium chloride.

**Table 6:** Correlation between lesion size or proportion lesion and performance in LiCl-injected rats

		Correlation statistics					
		Taste-visceral pairing		TR retention		Two-bottle test	
		Total ING	Total AVS	Total ING	Total AVS	24 h	48 h
Overall lesion	*r*	0.3857	−0.4145	0.07662	−0.05735	0.4613	0.3495
	*p*	0.0842	0.0618	0.7413	0.8050	0.0307*	0.1108
IC_2_+IC_3_	*r*	0.3143	−0.4770	0.1364	−0.2274	0.5528	0.4218
	*p*	0.1653	0.0288*	0.5556	0.3214	0.0076**	0.0506
IC_2_	*r*	0.3655	−0.3776	0.1094	−0.1043	0.4394	0.3596
	*p*	0.1033	0.0915	0.6367	0.6528	0.0407*	0.1003
Anterior half of IC_2_	*r*	0.3425	−0.3480	0.1239	−0.1358	0.3628	0.2455
	*p*	0.1286	0.1222	0.5925	0.5571	0.0970	0.2709
Posterior half of IC_2_	*r*	0.2990	−0.4456	0.1368	−0.1984	0.4994	0.3845
	*p*	0.1879	0.0429*	0.5543	0.3885	0.0180*	0.0773
IC_3_	*r*	0.1786	−0.3077	0.05716	−0.1356	0.3716	0.2926
	*p*	0.4385	0.1748	0.8056	0.5578	0.0886	0.1864
Anterior half of IC_3_	*r*	0.1700	−0.3164	0.07296	−0.2073	0.4604	0.3777
	*p*	0.4612	0.1622	0.7533	0.3673	0.0311*	0.0831
Posterior half of IC_3_	*r*	−0.01301	−0.07670	−0.08203	0.06534	0.1081	0.09451
	*p*	0.9554	0.7411	0.7237	0.7784	0.6321	0.6757

Spearman’s rank-order correlation was conducted (*r*: Spearman’s correlation coefficient, *p*: statistical significance); *p* values with an asterisk (*) indicate statistically significant correlations. IC, Insular cortex; Ing, Ingestive; Avs, Aversive.

#### Extensive lesions in IC_2_+IC_3_ attenuate the subsequent avoidance of a substance associated with LiCl

##### TR test

**Figure 5.**
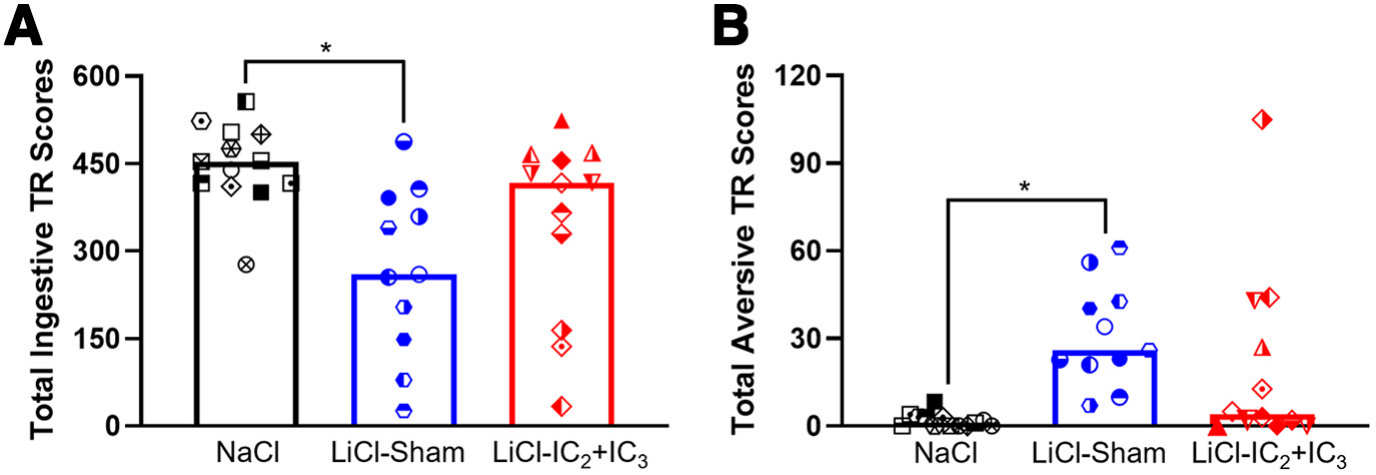
Lesions in IC_2_ and IC_3_ attenuated, but did not completely prevent, conditioned taste aversion on the TR retention test. Median cumulative ingestive (***A***) and aversive (***B***) TR scores in response to 30-s IO sucrose infusions during retention are plotted with different symbols for individual animals in NaCl-injected group (*n* = 13), LiCl-injected sham-operated control group (*n* = 11), and LiCl-injected IC_2_+IC_3_ lesion group (*n* = 12). Statistical significances are adjusted based on Benjamini–Hochberg false discovery rate for multiple comparisons and indicated as asterisks. Statistics are in Table 7. Ingestive and aversive TR scores in this retention test are plotted as a function of time following the intraperitoneal injection of either NaCl or LiCl in Extended Data Figures 5-1, 5-2, and 5-3. IC, Insular cortex; TR, Taste reactivity; LiCl, Lithium chloride; NaCl, Sodium chloride.

Next, we assessed whether rats with IC_2_+IC_3_ lesions who previously received IO sucrose paired with LiCl acquired conditioned responses to the orosensory CS. To do this, all rats were tested for their taste-guided responses to IO sucrose starting 20 min after an intraperitoneal injection of LiCl. This design was based on a prior study that showed rats do not acquire an aversion to an IO-infused stimulus if the negative visceral state precedes the first IO infusion by at least 20 min (Spector et al., 1988). In other words, rats only acquire a CTAver when the IO infusate is experienced before and/or during the onset of the LiCl-induced malaise. Second, this same study found that even neurally-intact rats display a relatively weak conditioned aversion to IO sucrose after the single serial TR pairing session. But, if the conditioned rats are put back into the negative visceral state produced by LiCl (by injecting LiCl 20 min before IO infusion), then aversive responding is significantly bolstered. Therefore, here, all rats were primed with LiCl 20 min before the IO infusions to determine whether the LiCl-IC_2_+IC_3_ lesion group displayed weaker aversive responses than the LiCl-Sham group on the postpairing test. Indeed, the Sham-operated rats previously treated with LiCl maintained significantly reduced total ingestive response and increased total aversive response to IO sucrose, as compared with the NaCl-injected control rats (Fig. 5*A*,*B*; Table 7), showing that the previous sucrose-LiCl pairing yielded a learned taste aversion. The LiCl-injected group with IC_2_+IC_3_ lesions, on the other hand, displayed high total ingestive and low total aversive TR to IO sucrose, which did not statistically differ from that of the NaCl-treated control rats or the LiCl-treated Sham rats. In fact, whereas the LiCl-injected Sham rats displayed consistent reductions in ingestive TR and increases in aversive TR across the retention test, the LiCl IC_2_+IC_3_ rats only showed significant reduction in ingestive TR at minute 30 (Extended Data Fig. 5-1). We noted that a subset of animals in LiCl IC_2_+IC_3_ lesion group appeared to be normal in terms of shifting their taste responses to sucrose highlighting the behavioral heterogeneity within this group. Yet, neither total lesion size nor the extent of lesion within any of the IC subregions of interest analyzed was found to be associated with TR on this retention test (Table 6).

**Table 7:** Comparison of total ingestive or aversive TR score between groups on the retention test

Total ingestive score, q* = 0.0167			
	Na	Sham-Li	IC_2_+IC_3_-Li
Na		0.0004+	0.1728
Sham-Li			0.1321
Total aversive score, q* = 0.0167			
	Na	Sham-Li	IC_2_+IC_3_-Li
Na		<0.0001+	0.0712
Sham-Li			0.0764

Corresponds to Figure 5*A,B*. Significance level was adjusted based on Benjamini–Hochberg false discovery rate for multiple comparisons (q*; Benjamini and Hochberg, 1995). Values with a plus symbol (+) are statistically significant after correction. IC, Insular cortex; Li, Lithium chloride; Na, Sodium chloride.

10.1523/ENEURO.0527-21.2022.f5-2 Extended Data Figure 5-2 Comparison of ingestive TR scores between groups at select time points across the retention test. Corresponds to Figure 5. Download Figure 5-2, DOC file.

10.1523/ENEURO.0527-21.2022.f5-3 Extended Data Figure 5-3 Comparison of aversive TR scores between groups at select time points across the retention test. Corresponds to Figure 5 and Extended Data Figure 5-1. Download Figure 5-3, DOC file.

10.1523/ENEURO.0527-21.2022.f5-1 Extended Data Figure 5-1 A, B, Median (+ Semi-IQR) ingestive and aversive TR scores to IO sucrose infusions in retention session are plotted as a function of time following the intraperitoneal injection of either NaCI (n = 13) or LiCI (n = 11 for sham, n = 12 for IC2+IC3 lesion; black arrow). Open symbol is assigned for LiCl-injected groups at each timepoint if there is statistical significance (p ≤ 0.05) compared to the NaCl group. Statistics are in Extended Data Figures 5-2 and 5-3. Download Figure 5-1, TIF file.

##### Two-bottle avoidance test

**Figure 6.**
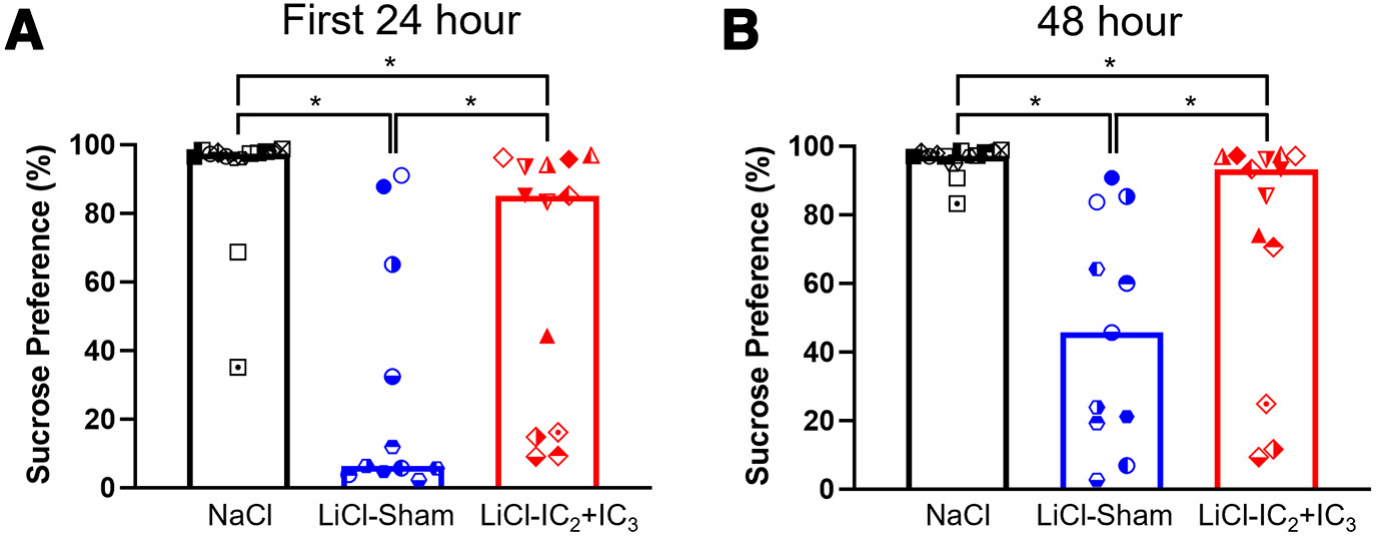
Lesions in IC_2_ and IC_3_ impaired CTAvoid on the two-bottle choice retention test. Median percent sucrose preference over water during the first 24-h (***A***) or total 48-h (***B***) two-bottle choice test are plotted with different symbols for individual animals in NaCl-injected group (*n* = 14), LiCl-injected sham-operated control group (*n* = 11) and LiCl-injected IC_2_+IC_3_ lesion group (*n* = 13). Statistical significances are adjusted based on Benjamini–Hochberg false discovery rate for multiple comparisons and indicated as asterisks. Statistics are on Table 8. Median values of sucrose intake (g) during the first 24 or 48 h are plotted in Extended Data Figure 6-1, with statistics in Extended Data Figure 6-3. Two subgroups of LiCl-injected IC_2_+IC_3_ group with the greatest and the least impairment were compared for their lesion sites in Extended Data Figure 6-2. IC, Insular cortex; TR, Taste reactivity; LiCl, Lithium chloride; NaCl, Sodium chloride.

Next, all rats were tested for their avoidance of sucrose in an *ad libitum* two-bottle choice test (vs water) in the home cage. In the first 24 h, the NaCl-injected control group preferred sucrose to water, whereas the LiCl-injected Sham group largely avoided sucrose (Fig. 6*A*; Table 8; Extended Data Fig. 6-1*A*). During the first 24 h and across the 48-h test, the LiCl-injected rats with IC_2_+IC_3_ lesions actually preferred sucrose, with a preference score that was significantly higher than that of their Sham counterparts. Similar patterns were observed over the full 48 h of the test (Fig. 6*B*; Table 8; Extended Data Figs. 6-1*B*, 6-3). Overall lesion size was positively correlated with 24-h sucrose preference, and this appeared to be mainly because of the extent of neuronal loss across IC_2_ and IC_3_, especially in the posterior IC_2_ and anterior IC_3_ subregions. Conditioned sucrose avoidance of LiCl-treated IC_2_ or IC_3_ groups is shown for comparison in Extended Data Figures 4-2*D* and 4–3*D*.

**Table 8:** Comparison of sucrose preference between groups on the two-bottle choice test

First 24 h, q* = 0.05			
	Na	Sham-Li	IC_2_+IC_3_-Li
Na		<0.0001+	0.0005+
Sham-Li			0.0088+
Full 48 h, q* = 0.05			
	Na	Sham-Li	IC_2_+IC_3_-Li
Na		<0.0001+	0.0045+
Sham-Li			0.0183+

Corresponds to Figure 6. Significance level was adjusted based on Benjamini–Hochberg false discovery rate for multiple comparisons (q*; Benjamini and Hochberg, 1995). Values with a plus symbol (+) are statistically significant after correction.

10.1523/ENEURO.0527-21.2022.f6-2 Extended Data Figure 6-2 Comparison of lesion sites between two subgroups of LiCl-injected animals with IC2 and IC3 lesions. Group-wise overlap lesion maps showing the average lesion scores in color-coded manner on 2D lesion mapping grids are presented for the rats in LiCl-IC2+IC3 group with the greatest impairment (“Top 5”; individual symbols: ◇◭⧩◆◮) and those with the least impairment (“Bottom 4”; individual symbols: ⬗⬘⬙⟐) in the first 24 h of the two-bottle choice test. Solid and dotted lines indicate different AP levels relative to bregma including IC2 borders (+1.2 and +0.2 mm) and IC3 borders (+0.2 and –0.8 mm). A, anterior to bregma, P, posterior to bregma, D, dorsal to the granular layer (GI); GI, granular IC; DI, dysgranular IC; AI, agranular IC (dorsal to the rhinal fissure); V, ventral to rhinal fissure. Download Figure 6-2, TIF file.

10.1523/ENEURO.0527-21.2022.f6-1 Extended Data Figure 6-1 Animals with IC2 and IC3 lesions given LiCl at the initial pairing failed to avoid sucrose consumption on the two-bottle choice retention test. Median values of sucrose intake (g) during the first 24 h (A) or 48 h (B) in two-bottle test are plotted with different symbols for individual animals in NaCl group (n = 14), LiCl-sham control group (n = 11) and LiCl-IC2+IC3 lesion group (n = 13). Different letters above the bars indicate statistical difference (p ≤ 0.05) between groups. Statistics are on Extended Data Figure 6-3. Download Figure 6-1, TIF file.

10.1523/ENEURO.0527-21.2022.f6-3 Extended Data Figure 6-3 Comparison of sucrose intake between groups on the two-bottle choice test. Corresponds to Figure 6 and Extended Data Figure 6-1. Download Figure 6-3, DOC file.

#### Individual rats with IC_2_+IC_3_ lesions that exhibit impaired avoidance generally display impairments on the prior measures of taste aversion

**Figure 7.**
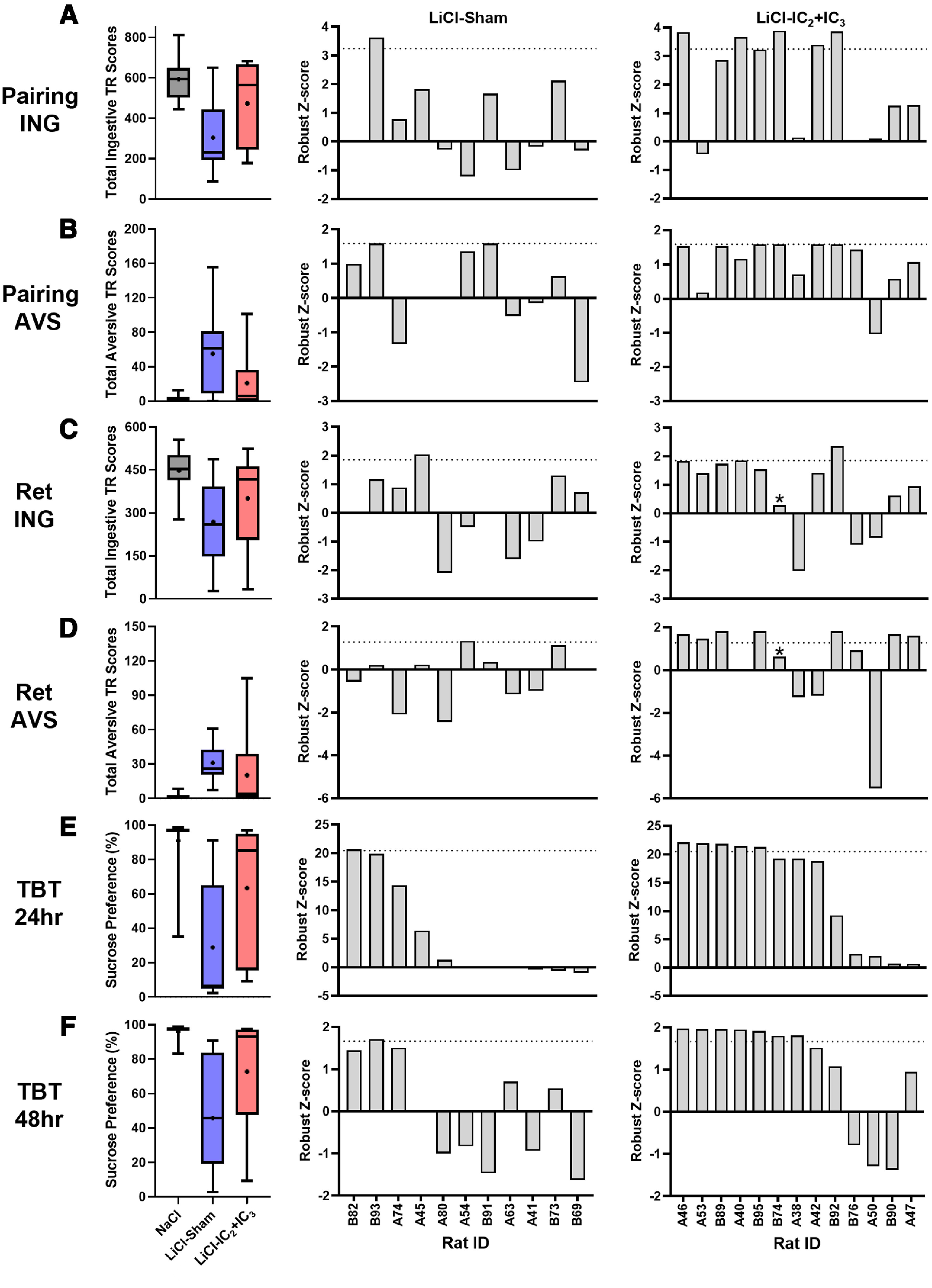
Group performance comparison and standardized behavior scores of individual animals in LiCl-given groups. Group-wise comparison (left) and individual animals’ performance in LiCl-sham group (middle) and LiCl-IC_2_+IC_3_ group (right) for total ingestive (***A***) and aversive TR (***B***) in the stimulus pairing session, total ingestive (***C***) and aversive TR (***D***) in retention and sucrose preference during the first 24 h (***E***) and 48 h (***F***) in two-bottle test. Left column, Box and whisker plots of TR scores or sucrose preference scores for all NaCl-injected or LiCl-injected groups included in the statistical analyses on each of the main behavioral measures (top to bottom). The + within each box represents the group mean. Each group is labeled at the bottom of ***F***. Middle and right columns, The animals are arranged in order of high to low sucrose preference in 24-h two-bottle test and the robust Z scores of individual animals in each test are plotted to visualize their performance. Their IDs are denoted on the *x*-axis at the bottom of ***F***: the IDs of animals in phase 1 start with “A” and the animals in phase 2 start with “B.” For aversive TRs (***B***, ***D***), the signs of Z scores are reversed so that the positive values indicate more impairment in their performance. Asterisk above the bar indicates incomplete data. Ret, retention; TBT, two-bottle test; ING, ingestive TR; AVS, aversive TR. In Extended Data Figure 7-1, LiCl-given animals with IC_2_ or IC_3_ lesion are included and standardized behavior scores of all LiCl-given lesion animals are plotted. IC, Insular cortex; TR, Taste reactivity; LiCl, Lithium chloride; NaCl, Sodium chloride.

To examine behavioral patterns across the taste-visceral pairing and retention tests of individual rats, group median performance across each phase is displayed in Figure 7, left column, with the standardized behavioral scores of individual LiCl-treated rats in Sham or IC_2_+IC_3_ lesion groups in Figure 7, middle and right-hand columns. The rats in each group were sorted according to their robust Z-score on the first 24-h two-bottle test (high/most impaired to low/least impaired) and plotted accordingly (left to right) for each behavioral measure. The LiCl IC_2_+IC_3_ rats that were significantly impaired on the two-bottle test tended to be impaired on earlier measures including TR on the pairing and retention tests (Fig. 7, right column), while no such systematic pattern was seen in the LiCl Sham group (Fig. 7, middle column).

10.1523/ENEURO.0527-21.2022.f7-1 Extended Data Figure 7-1 Standardized behavior scores of LiCl-given animals with IC2 and/or IC3 lesion. The robust Z-scores of LiCl-injected lesion animals on ingestive (A) and aversive TR (B) in the stimulus pairing session, ingestive TR (C) and aversive TR (D) in retention and sucrose preference over water during the first 24 hour (E) and 48 hour (F) in two bottle test are plotted. For aversive TRs, (B) and (D), the signs of Z scores are reversed so that the positive values indicate more impairment in their performance. The animals are arranged in order of showing high to low sucrose preference in 24-hour two bottle test and their IDs are denoted on the x axis in (F). Animals in phase 1 start with ’A’ and animals in phase 2 start with ’B’. Bar appearance is different for each lesion group (IC2: open, IC3: hatched, IC2+IC3: filled with gray color). Asterisk above the bar indicates incomplete data. Abbreviations: RET, retention; TBT, two bottle test; ING, ingestive TR; AVS, aversive TR. Download Figure 7-1, TIF file.

## Discussion

Schier et al. (2014) showed that the posterior part of IC, encompassing the posterior gustatory zone and the anterior visceroceptive zone (IC_2_+IC_3_), is required to properly avoid solutions previously paired with the emetic agent, LiCl. In particular, the deficit was most consistently found in the rats that had lesions spanning the entire IC_2_ region (Schier et al., 2016). The precise process(es) disrupted by posterior IC lesions to produce such a deficit, however, remained unclear. The present findings showed that, by and large, lesions in the IC_2_+IC_3_ region interfered with the ability to rapidly express changes in taste-guided behaviors to sucrose in response to the coincident aversive effects of LiCl. This, in turn, generally weakened the ability to later avoid the sugar in the two-bottle choice test conducted over 24 and 48 h in the home cage. Taken together, these results provide novel evidence that the IC_2_+IC_3_ region contributes to isochronous adjustments to taste-guided behaviors according to the change in visceral state, which may contribute to subsequent deficits in CTAvoid.

### The roles of IC_2_+IC_3_ in conditioned taste aversion

The failure to express a conditioned taste aversion/avoidance could be related to deficits in primary sensory processing, including for the taste CS and/or interoceptive US, integrative processing, memory, executing a response or some combination thereof (Fig. 1; Spector et al., 1988). The fact that lesions in IC_2_+IC_3_ generally perturbed the ability to progressively shift behavioral responses in accord with the emergence of a negative interoceptive state in the serial TR test, which has minimal, if any, memorial demands narrows the search for mechanism to the processes at work during the initial taste-visceral pairing session. Prior work showed that rats with lesions encroaching IC_2_ are not ageusic to suprathreshold concentrations of sucrose and show normal unconditioned oromotor and somatic responses to sucrose, though whether more posterior lesions would produce taste deficits in these contexts is unknown (Bales et al., 2015; King et al., 2015). Thus, three main possibilities remain. The delay to suppress ingestive TR and/or express aversive TR after the initial intraperitoneal injection of LiCl could reflect either a blunted sensitivity to the visceral stimulus or a failure to integrate the two sources of sensory input to make the adaptive responses. Prior studies found that rats with bilateral lesions in IC_2_ and/or IC_3_ were slower to acquire a learned taste avoidance than the LiCl-injected Sham and Unilateral lesion controls (Schier et al., 2014, 2016). Although the animals with these lesions did ultimately acquire learned taste avoidance in a short-term intake test after two stimulus pairings, it was tenuous at best for a subset of rats with extensive IC_2_ lesions (Schier et al., 2016). That is, after demonstrating competence in avoiding the CS in short (15 min) single bottle test, these rats ultimately failed to avoid consuming the CS in a longer, 48-h, two-bottle test. The slower initial learning and the expedited extinction could stem from diminished interoceptive and/or CS-US integrative processing. That being said, Schier et al. (2014, 2016) also found that lesions in this IC region interfered with the ability to avoid a solution that was associated with LiCl before the lesion. Thus, at the very least, the contribution of this region to CTAvoid is not limited to real time viscerosensory and/or integrative processes.

A parsimonious possibility is that this region is involved in expressing adaptive taste-guided behaviors in the context of ongoing interoceptive and/or associated events. The decision to consume or not to consume a particular substance involves many factors, such as weighing the cost-benefit of that action against the current physiological state, other competing drives, other resources, the food’s conditioned/unconditioned reward value, and the predicted consequences of its consumption. The types of deficits associated with loss of function in IC_2_+IC_3_ region could stem from a weakened ability to flexibly switch hardwired, unconditioned taste-guided behaviors in light of discrepant physiological or experiential sources of information. A recent study (Livneh et al., 2020) found that the activity of some IC neurons reflects not only the current physiological status like water or energy depletion, but corresponds to the expected consequences of cues associated with water or food. This supports the hypothesis that insular cortical neurons might be involved in integrative function of current status with cue-predicted outcomes. It is possible that the lesions encompassing IC_2_+IC_3_ interrupted such function of IC, which led to impaired performance in the present study in terms of the ability to shift toward rejective responses under the influence of the LiCl injection. Such a deficit could also explain why, in the previous studies, animals with lesions in the IC_2_ region were unable to avoid a solution that was associated with LiCl before the lesion (Schier et al., 2014, 2016). It will be important to determine whether such a role is specific to LiCl-based conditioned taste aversion or it extends to other types of ingestive behaviors or indeed other types of USs (Geddes et al., 2008; Schier et al., 2019). After all, associative pairings are known to render taste-responsive neurons in the GC responsive to the predictive stimulus and silencing GC during the decision phase of a taste-guided two response operant choice task, but not during the taste stimulus itself, significantly disrupted correct response selection (Vincis and Fontanini, 2016; Vincis et al., 2020).

The association of a taste stimulus with LiCl-induced malaise can cultivate two different types of learned responses: conditioned taste aversion and/or avoidance (Pelchat et al., 1983; Parker, 2003; Schier et al., 2019; Schier and Spector, 2019). Because previous studies examining the role of posterior IC in CTA used intake measurements, it had remained unclear whether the lesion-induced impairments were related to a diminished ability to avoid consuming a taste solution that was associated with LiCl (appetitive or goal-directed deficit) or a failure to alter taste-guided oromotor and somatic repertoires (consummatory deficit). In general, rats with IC_2_+IC_3_ lesions in the present study showed deficits in both domains. The fact that these rats with cortical lesions displayed a diminished capacity to shift their taste-guided reactions in the initial taste-LiCl pairing session suggests that this region of IC may play an important role in acutely revising responses linked to hedonic evaluation. One question that still remains is whether the failure to avoid the taste CS in the two-bottle test was because of this consummatory deficit or whether there were additional appetitive deficits produced by the lesion. The types of behavioral deficits observed here generally recapitulate those observed following lesions of the gustatory zone of the parabrachial nucleus (gPBN) using a similar paradigm (Spector et al., 1992; Reilly et al., 1993; Grigson et al., 1998). Provided gPBN sends projections to IC via the ventropostero-medial nucleus of the thalamus, it seems likely that these two regions form a key circuit underlying taste-interoceptive integration. Future work targeting these gPBN to IC projections in a specific manner will be illuminative. Moreover, given previous studies have shown impaired avoidance of a tastant that was paired with LiCl before the lesion, future studies should investigate whether IC_2_+IC_3_ is required to express taste-guided consummatory changes under these conditions.

### Sources of variability

There was a considerable amount of behavioral variability, even within our LiCl-injected Sham group, in terms of the extent to which sucrose-elicited TR was shifted or sucrose preference was blunted in the present study. The serial TR paradigm used here was designed to allow us to observe ongoing changes in taste-guided behaviors in response to the gradual onset of a LiCl-induced toxicosis. The trade-off is that some of these task parameters, like limiting taste stimulation to ten ∼30-s presentations, using a submaximal LiCl dose, providing only a single taste-visceral pairing session, and shifting interoceptive and physical contexts between conditioning and testing, would be expected to curtail associative learning or sustained expression of that learning. Nevertheless, on a group-wide basis, the approach revealed significant differences among rats with IC_2_+IC_3_ lesions and the Sham controls.

Even within our group of rats with confirmed extensive lesion in both IC_2_ and IC_3_, we had a subset that was severely impaired and a subset that was essentially unaffected by the lesion. In general, this did not appear to be test-dependent. In other words, the LiCl-injected rats with IC_2_+IC_3_ lesions that were deemed impaired in their ingestive or aversive TR in the initial pairing session were more or less impaired on the remaining test sessions (Fig. 7, right column). This overall effect was even more pronounced when the behavioral data of LiCl-treated rats with lesions only in IC_2_ or IC_3_ are also considered (Extended Data Fig. 7-1). Nevertheless, lesion size and/or topography may contribute to the variability in deficits. Schier et al. (2016) noted that the “lesion hotspot” was most commonly found in IC_2_. Interestingly, we had a subset of rats in the present study that had massive lesions in IC_2_, but these rats were not overtly impaired on any of our measures. Moreover, we had a subset of rats with extensive lesion in IC_2_ only, and although the small sample size precluded statistical comparisons, qualitatively speaking this group appeared competent on these various behavioral measures (Extended Data Fig. 4-2), as did rats with extensive lesions limited to IC_3_ (Extended Data Fig. 4-3). Correlational analyses (Table 6) indicated that the proportion of IC_2_+IC_3_ and posterior IC_2_ with lesion were significantly negatively correlated with both aversive TR in the initial pairing session and sucrose preference in the 24-h two-bottle retention test. The proportion of lesion in anterior IC_3_ was significantly negatively related to sucrose preference in the 24-h two-bottle retention test. Lesion size in anterior IC_2_ or posterior IC_3_ was not significantly correlated with any behavioral performance. In addition, we compared the lesion sites of two subgroups from the LiCl-IC_2_+IC_3_ group showing the greatest impairment (“Top 5”) and the least impairment (“Bottom 4”) in the 24-h two-bottle avoidance test. While they both had a full lesion in IC_2_-IC_3_ border, we noticed that “Top 5” had more complete lesion in IC_2_ compared with the “Bottom 4” (Extended Data Fig. 6-2), perhaps especially in GI and AI. Therefore, we conclude that, in general, extensive lesion in IC_2_, including the posterior IC_2_ and IC_2_-IC_3_ border, is required to induce the functional deficits we observed. We cannot exclude the possibility that there are simply individual differences in the functional topography of the IC such that a lesion in the same anatomic location interferes with adaptive taste-guided responding in one rat, but not in another. More studies are needed to refine the specific area, neuronal subtypes, and/or extent of lesion in IC sufficient to disrupt CTA and related processes.

In the present study, loss of function in the posterior IC (IC_2_+IC_3_) severely disrupted the ability to modify taste-guided oromotor and somatic reactions in the face of an ongoing change in visceral state produced by LiCl and resulted in an impaired ability to avoid the associated taste stimulus in subsequent retention test. The results provide new insights into the neural organization of taste-visceral integrative processes that underlie conditioned taste aversion and avoidance.

10.1523/ENEURO.0527-21.2022.f4-5 Extended Data Figure 4-5 Comparison of the NaCl-injected groups on each behavioral test. Corresponds to Figures 4–6. Download Figure 4-5, DOC file.

## Synthesis

Reviewing Editor: Arianna Maffei, Stony Brook University Decisions are customarily a result of the Reviewing Editor and the peer reviewers coming together and discussing their recommendations until a consensus is reached. When revisions are invited, a fact-based synthesis statement explaining their decision and outlining what is needed to prepare a revision will be listed below. The following reviewer(s) agreed to reveal their identity: Chad Samuelsen.

The reviewers agreed that the study addresses and important question regarding the role of the insular cortex in aversive behavior to taste stimuli. However they raised substantial concerns regarding the presentation of the data, the insufficient statistical analysis and the description of the methods.

Figures would benefit from a more coherent choice of plots and clearer application of statistical analysis would make the data more compelling. It is unclear why for certain data the authors used bar plots and in other figures used box and whisker plots, for example.

In addition, certain experiments and analysis lacked a rationale. It appears that the analysis was performed on a subset of the data and not on the entire dataset, for example responses to only a subset of infusions are included.

Finally, the reviewers agreed that several statements, including the main conclusion of the study and the comparison across lesion groups, lack sufficient statistical support.

The detailed comments of the reviewers are reported below.

Reviewer #1

This manuscript describes a series of experiments to test whether the posterior insular cortex is necessary for the expression of taste aversions, in addition to the known role of this region in expressing taste avoidance. The authors address this question by subjecting rats with lesions to the IC to multiple sets of behavioral tests assessing taste aversion and taste avoidance. Lesions were mapped using a highly detailed lesion mapping approach. The introduction is well-written and the overall question is clear. However, the Methods section lacks important detail, and the presentation of results lacks structure and quantification. Most important, the main conclusion of the paper is not supported by the data, as the critical comparison did not yield significant results (TR scores did not differ between lesion version sham LiCl groups).

Major:

The description of the lesion mapping procedure raises many unaddressed questions:

- The term “grid cell” (or “cell” later on in the text) is used to refer to coordinates. I find this term confusing given the more common use of the word “cell” to refer to a biological unit of tissue. I suggest using “pixel” or “X-Y coordinate” to avoid confusion.

- On the basis of what observation exactly is damage scored? What does 100%/50% damage refer to in terms of the tissue.

- The status of the area marked “ventral to the rhinal fissure” is unclear to me. Judging from the example figures, it appears to include agranular insular cortex, but in the text the phrasing “ventral to the insular cortex” is sometimes used (e.g., section “Lesion mapping” in the Methods).

- Images of tissue are unclear and should be shown at higher magnification/resolution. In Supplementary Figure 6 (-0.8, left), for example, there appears to be damage in the V region, but none is indicated in the lesion map.

- Are the dorsal and ventral regions included in “regions of interest”? What about the claustrum and region medial to the claustrum?

- The procedure for aligning lesion maps across animals is not clear to me. What does it mean to “round the AP scale to the closest multiple of 10”?

- What is meant by “AP thickness”? (section Lesion Mapping)

- What are the two landmarks referred to? (section Lesion Mapping)

The use of the TR retention test is unclear to me. Is it meant to assess the strength of the aversive memory? It seems ineffective in doing this, because the finding that the animals previously injected with LiCl showed a stronger negative response to sucrose compared to the other groups could simply reflect latent inhibition in the other groups.

Only data from a subset of infusions is reported, please report entire time course. Splitting the infusions up into 2 epochs (early and late) and comparing the results between experimental groups for each epoch may provide more statistical power.

Data from the IC2 or IC3 lesion rats is plotted and referenced without any quantification. Specific questions regarding these groups along with quantitative evaluation of these questions is necessary if data from these animals were to be included in the manuscript.

The use of correlation is not well-justified. First, visual inspection of the behavioral score distributions suggests that they may be bimodal rather than continuous. Second, a more fine grained analysis of the lesions that takes into account subregions identified by the elaborate lesion mapping procedure may provide a better explanation of these results than overall lesion size. For example, 6 animals in the LiCl-IC2+IC3 group in Figure 3C appear to be unaffected by their lesions; what is unique about these?

What is the rationale for the analysis in Figure 6? Data lack quantitative evaluation. Caption appears to be missing text.

Minor:

Introduction

The distinction between “sensory” and “visceral” is not intuitive, as gustatory input is sometimes referred to as visceral, and visceral input is generally referred to as sensory. Might be more useful to refer to the distinct biological sources of the inputs evoked by the US and CS.

The term “oromotor and somatic responses” to refer to TR behavior is unclear; do you intend it to include both oral and somatic motor responses?

What is meant by “online (emphasis) integration"; does it not simply refer to step 3 in Figure 1?

Methods

How much total lost time was present in the videos?

Results

Overall, it would help to have the presentation of results guided by explicit questions, allowing for a better judgement of the appropriateness of the approach and statistical evaluation.

In Figures 3-5, it is unclear to me how exactly the letters indicate significant differences between groups. It should be relatively simple to incorporate all the relevant information from Tables 2-6 into these figures, for example by using common brackets and asterisks (rendering the tables unnecessary).

Reviewer #2

This report provides novel insights into the taste-visceral interactions of the insular cortex. The authors explore how loss of function in a subregion of insular cortex (via ibotenic acid lesions) influences taste-related oromotor and somatic consummatory behaviors in response to a negative visceral event (i.e. LiCl-induced malaise). The authors measure both ingestive and aversive consummatory behaviors (in real-time) during the intraoral delivery of sucrose after LiCl injection in rats with and without lesions of a subregion of insular cortex. Consistent with prior reports, the authors show that lesions of this region disrupt conditioned taste avoidance and severely disrupts behavioral modification of taste-dependent oromotor and somatic reactions during negative changes in the visceral state.

The experimental findings and authors interpretations are quite interesting. However, I believe their findings would be more effectively communicated by modifying how the data presented in the figures and adding some explanation to certain aspects of the experimental design.

Title:

1. The authors should consider adding the species to the title.

Methods:

1. Why were different volumes of iobtenic acid used for the surgeries in Phase 1 (0.18 μl) and Phase 2 (0.1 μl)? Were there any significant differences in the size and breadth of the lesions between these two surgical groups?

2. Please revise the sentence explaining the robust Z-score standardization. “In order to examine patterns of behavior across the multiple behavioral assessments, each individual rat’s behavior performance was converted to a robust Z-score standardized against the median and deviation of the LiCl Sham group the main behavioral measurements.”

3. I appreciated the authors method of correcting for the portions of trials when data could not be collected due to obscured views. However, I believe a couple of points of clarification would help with communication. The author’s description says “The ingestive and aversive TR scores from the videos that had >1 second of no data were corrected for lost time by dividing the total scores by scorable time and then multiplying that by 30.” First, were the ingestive and aversive TR scores summed (i.e. total scores) then divided by the scorable time or was the total ingestive TR score divided by the scorable time and the total aversive TR score divided by the scorable time? Second, I hate to admit but it took me far too long to understand why the scores were then multiplied by 30. The authors may wish to clarify here that this corresponds to the length of time of a single infusion trial.

4. Why did the authors choose to sum each rats Ingestive TR scores and Aversive TR scores across the 4 taste-visceral time-points and the 3 retention time-points? Wouldn’t finding the mean score for each rat per session allow for direct comparisons in Ingestive TR scores and Aversive TR scores between the two sessions?

Results Section:

1. I appreciate that the author’s provided tables showing the statistical differences for each experimental figure because the graphs were very confusing. For example, Figure 3C and 3D list letters (a; b; a,b) above the bar graphs with the caption saying “Different letters above the bars indicate a statistical difference (p<0.05) between groups.” However, according to the text and Table 4, the only significant difference for both the Ingestive Score and the Aversion Score was for the comparison between the NaCl group (written as Na instead of NaCl on the table) and the Sham-Li group. This same nomenclature is used for Figures 3CD, 4AB, and 5AB. Using black bars and asterisks to indicate significant differences would likely be easier to follow.

2. Relatedly, Figures 3CD, 4AB, and 5AB show each rats score with a bar graph at the median. However, the Semi-IQR is only shown when these plots are recreated in Figure 6. Furthermore, I understand that the mean and SEM are heavily influenced by outliers, but plotting the data as the median {plus minus} SIQR masks the variability of the data. The authors should consider remaking each figure as a box and whisker plot and indicate the mean for each group (with a plus sign for example) as well.

3. I appreciate the authors for providing the data as supplemental material for the incomplete groups of rats with either IC2 or IC3 lesions. For the most part, the authors are careful to refer to this supplementary information as a general “for comparison” (e.g. at the end of Results sections, Extensive Lesions in IC2+IC3 disrupted taste-guided oromotor and somatic responses to sucrose following IP LiCl injections and Two-Bottle Avoidance Test). However, they also use this incomplete data set (which they say were not used for analyses “due to the limited sample sizes.”) to directly compare with results from the complete IC2+IC3 data set at the end of Results sections, Taste Reactivity Test and Individual Rats with IC2+IC3 lesions that exhibit impaired avoidance generally display impairments on the prior measures of taste aversion. These statements should be revised to remove any interpretation of the incomplete data set to mirror the more general “for comparison” statements. Of course, the authors are free to move these points to the Discussion section if they wish to discuss how the results of the incomplete data set shape their interpretations of the IC2+IC3 data set.

4. Unfortunately, I was unable to completely evaluate Figure 6 because the caption was cut off. I’m sure I would be better able to grasp the details of the middle and right columns with the complete figure caption.

5. What do the “r” and “p” stand for in Table 7?

## Author Response

We greatly appreciate the overall positive reception of our manuscript, as well as the insightful and constructive feedback from the editors and reviewers. We have carefully considered each of the points and have modified the manuscript to best address the issues raised. We provide justification for cases in which we did not make a recommended change. A point-by-point response to the concerns raised in the prior review are detailed below. We feel the quality of the manuscript has been significantly enhanced through these revisions and we hope that you will find them acceptable.

REVIEWER 1

Major

1. The term “grid cell” (or “cell” later on in the text) is used to refer to coordinates. I find this term confusing given the more common use of the word “cell” to refer to a biological unit of tissue. I suggest using “pixel” or “X-Y coordinate” to avoid confusion.

Response: We understand the concern, but we hesitate to use the word “pixel” or “X-Y coordinate” which may confuse the readers as well (e.g., “X-Y coordinates” can be confused with lesion “A-P coordinates”). The maps are generated with MS Excel ®, which uses the “cell” terminology, and this term has been used in prior publications using a similar mapping system. Nevertheless, we revised the text to use “grid cell” each time we refer to the maps to minimize the confusion. These changes are indicated in the red bold text throughout the Lesion Mapping section of the Materials and Methods.

2. On the basis of what observation exactly is damage scored? What does 100%/50% damage refer to in terms of the tissue.

Response: The thionin-stained brain sections were examined under a microscope by an experimenter blind to the surgery or treatment group of the animal. The area spanning the insular cortex was assessed for cellular loss. Accordingly, the extent of cell loss within the area represented by each grid cell was scored on a trinary scale: Red indicates the entire (100%) of corresponding region was lesioned, yellow indicates at least half, but less than 100% was lesioned, and white indicates that less than half of the corresponding region was lesioned. In order to make this clearer, we added some details in Lesion Mapping section under Materials and Methods (see lines 265-268).

3. The status of the area marked “ventral to the rhinal fissure” is unclear to me. Judging from the example figures, it appears to include agranular insular cortex, but in the text the phrasing “ventral to the insular cortex” is sometimes used (e.g., section “Lesion mapping” in the Methods).

Response: Yes, this is a good point. When the AP axis is anterior to 0.0 mm, all or some of the agranular division of insular cortex (AI) is above the rhinal fissure so this part of AI is not included in the area ventral to rhinal fissure (V). However, when the AP axis is posterior to 0.0 mm, AI falls below the rhinal fissure and is, therefore, included in the V column of our mapping grid. We revised the text to make this point clear in the Lesion Mapping section under Materials and Methods (see lines 260-264).

4. Images of tissue are unclear and should be shown at higher magnification/resolution. In Supplementary Figure 6 (-0.8, left), for example, there appears to be damage in the V region, but none is indicated in the lesion map.

Response: The photomicrographs in Figures 3-1 to 3-3 (previously Supplementary Figures 4-6) were re-imaged and magnified to be provided at the highest resolution possible and at a magnification to show the entire span of the lesion/or insular cortex. With respect to the issue raised in Figure 3-3 (previously Supplementary Figure 6), the V layer is primarily agranular cells, which are less dense than the overlying layers. For this reason, we agree that it can be difficult to differentiate lesion from normal tissue at without the aid of the microscope. Nonetheless, we appreciate that you brought this to our attention, because we realized that we did not adequately describe our criteria for rating damage to the dorsal and ventral subregions in the first submission. Essentially, for the regions above and below the gustatory and visceral insular cortices, to be considered at least 50% damaged, the lesion must span at least half the total height of the AI, DI, and GI layers above the rhinal fissure in that same brain section. As such, the image at -0.8 mm in the Figure 3-3 (previously Supplementary Figure 6) does not quite reach that criterion and a white grid cell was recorded for the V region. To clarify this, we added short description on how we delineated the D and V for mapping purposes in the Lesion Mapping section under Materials and Methods (see lines 260-264) and corresponding corrections have been made in Figures 1A-C and Figures 3-1 to 3-3 (previously Supplementary Figures 1, 4, 5 and 6).

5. Are the dorsal and ventral regions included in “regions of interest”? What about the claustrum and region medial to the claustrum?

Response: D, V, the claustrum, and the region medial to claustrum are not included in “regions of interest", but all are included in the total lesion size calculation. We now provide more detail about how the lesion scores are calculated (see lines 294-299).

6. The procedure for aligning lesion maps across animals is not clear to me. What does it mean to “round the AP scale to the closest multiple of 10”?

Response: We thank the reviewer for bringing our unfortunate lack of detail about this in the text to our attention. Briefly, serial 50 μm sections were collected for each brain. The lesion damage was recorded for each 50 μm section across successive grid rows in the Excel sheet. However, brains tend to shrink during the fixation process and the degree of shrinkage is not equal across all levels of the forebrain. Therefore, in order to compare the lesion maps across animals as accurately as possible, we used several key tissue landmarks (e.g., the joining of the corpus callosum, the joining of the anterior commissure, etc.) to anchor grid rows to various known AP levels across the brain (based on the Paxinos and Watson atlas, 6th edition). Then, to maximize precision for comparison across different brains, we figured the AP distance between each landmark, divided that by the number of rows occupied between the two landmarks for each brain, and then multiplied each row by that value. This essentially corrects the AP distance represented by each row. Then, each row distance was rounded to the nearest multiple of 10 microns (e.g., rounding 62 μm to 60 μm). Each row was further subdivided into 10 μm rows (in this example: 6 rows). This allowed us to put the individual symmetry maps on the same scale for alignment. We expanded our description of the lesion mapping system to clarify these steps (see lines 300-310).

7. What is meant by “AP thickness” (section Lesion Mapping)?

Response: We have replaced the term “AP thickness” to “AP distance represented by each row.”

8. What are the two landmarks referred to (section Lesion Mapping)?

Response: Multiple histological landmarks were chosen across the AP span of IC such as anterior start of the striatum, the joining of the corpus callosum, the rostral joining of the anterior commissure and more. Two adjacent landmarks were used to calculate the AP scale of each of the rows that are in between the two landmarks, as noted above, and now expanded upon in the text at lines 277-287.

9. The use of the TR retention test is unclear to me. Is it meant to assess the strength of the aversive memory? It seems ineffective in doing this, because the finding that the animals previously injected with LiCl showed a stronger negative response to sucrose compared to the other groups could simply reflect latent inhibition in the other groups.

Response: Yes, the TR retention test was conducted to quantitatively evaluate the animals’ responses to the reintroduced conditioned stimulus (CS; sucrose) which was previously presented with a negative unconditioned stimulus, US; LiCl) or neutral US (control; NaCl). In our TR retention test, LiCl was injected in all rats, irrespective of previous conditioning, 20 minutes before the first CS infusion. This was done to reinstate the general visceral conditions experienced by the group previously injected with LiCl during the taste-visceral US pairing session. This was based on the design on an earlier study. In that study, Spector et al. (1988) found that rats who had previously received the taste-LiCl pairing continued to display high ingestive responses to the CS in a retention test without LiCl on board but had reduced ingestive behaviors and elevated aversive behaviors in the retention test with LiCl on board. Moreover, they found that when the NaCl or LiCl injection preceded the first CS infusion by 20 minutes in the taste-visceral US pairing session, there was no significant difference in ingestive and aversive TR responses between the two injection groups in the retention test: The LiCl-injected group showed comparable conditioned TR responses to a control (NaCl-injected) group. Taken altogether, these findings suggested that the CS must precede the onset of the visceral US to foster CTA. Accordingly, we reasoned that if rats were not returned to the negative visceral state during the retention test, then we would essentially have a floor effect in our LiCl-sham group. This would effectively preclude our ability to determine if the Lesion LiCl were similarly affected by the previous CS-US pairing. Therefore, we opted to inject LiCl 20 minutes before the first CS infusion for all groups, because this appears to reveal expression of the CTA but itself is not sufficient to condition a taste aversion in order to maximize our ability to detect any lesion-based differences in conditioned behaviors. We greatly appreciate the reviewer for bringing the issue to our attention though. In addition to our brief explanation in the Methods (lines 195-197), we added more text in the Results section (lines 384-394) to explain the design and rationale.

10. Only data from a subset of infusions is reported, please report entire time course. Splitting the infusions up into 2 epochs (early and late) and comparing the results between experimental groups for each epoch may provide more statistical power.

Response: We indeed recorded during each IO infusion but chose to score a subset of time points that capture the dynamic phases of the responses (minute 5, 15, 25, and 35) based on Spector et al. (1988). We must admit that the manual counting of TR responses is very laborious and this study included 47 animals times 7 timepoints for a total of 329 files to process. Although we agree that splitting the infusions into early vs late epochs may increase the statistical power, we ultimately decided that presenting our data across time best captures the behavior of the animals, and their TR shifts from ingestive and aversive oromotor and somatic reactions to the oral sucrose infusions as the negative visceral event comes online. This allowed us to be sensitive to any delays in the shift across groups. We beg the reviewer’s indulgence on this issue because we are doubtful that any further scoring will provide significantly further clarity to the outcomes.

11. Data from the IC2 or IC3 lesion rats is plotted and referenced without any quantification. Specific questions regarding these groups along with quantitative evaluation of these questions is necessary if data from these animals were to be included in the manuscript.

Response: Quantitative evaluations of LiCl-injected IC2 or IC3 lesion groups were purposely excluded from the text and Figures 4-2 and 4-3 (previously Supplementary Figures 7-8) because of the low sample sizes. We opted to include the preliminary data from these smaller lesion groups sans analysis, because we believe it will enable the readers to do qualitative comparisons with other groups, especially the LiCl-IC2+IC3 double lesion group. We agree that the interpretation or comparison of incomplete dataset needs to be made carefully in the manuscript (see also: Reviewer 2, Results #3). Hence, we have thoroughly reviewed the manuscript and the relevant sentences were moved from Results section to General Discussion section (see lines 568-570 and 574-577).

12. The use of correlation is not well-justified. First, visual inspection of the behavioral score distributions suggests that they may be bimodal rather than continuous. Second, a more fine grained analysis of the lesions that takes into account subregions identified by the elaborate lesion mapping procedure may provide a better explanation of these results than overall lesion size. For example, 6 animals in the LiCl-IC2+IC3 group in Figure 3C appear to be unaffected by their lesions; what is unique about these?

Response: We tried to be as careful as possible with respect to drawing any strong conclusions related to the precise lesion location that produces the deficit. We did this for two reasons. First, we fully acknowledge and show the variability in the data, and we used non-parametric statistics, including Spearman’s Rank correlations. Not all rats that had complete IC2+IC3 lesions were universally impaired; some, as the reviewer points out and we pointed out in lines 406-409 and lines 563-570, essentially responded normally on select tests. We mapped the lesions with a very high level of spatial/topographical resolution. Our correlations were not only calculated for total lesion score, but for several subregions in and around IC (see Table 7). We pored over the maps and were unable to identify a specific feature of the lesions in impaired versus unimpaired rats for each test. We compared the group-wise overlap lesion maps from two subgroups in LiCl-IC2+IC3 rats showing the greatest impairment (’Top 5’; ◇◭⧩◆◮) and the least impairment (’Bottom 4’; ⬗⬘⬙⟐) in the 24-hour two-bottle avoidance test (Figure 6-2). While both subsets had {greater than or equal to} 50% lesions in IC2 and IC3 and a full lesion in IC2-IC3 border, the ’Top 5’ had more complete lesion in IC2 than the ’Bottom 4’. Therefore, we concluded that extensive IC2 lesion, including the posterior IC2 and IC2-IC3 border, is more predictive of the observed functional deficit. As we point out in the Discussion (beginning at line 566), we found that, in general, rats with IC2+IC3 lesions were impaired and that if an individual rat was impaired on one measure, it was generally impaired on other measures as well. Exceptions to this rule likely stem from minor variations in lesion placement that cannot be captured by the mapping system or from anatomical variability in functional topography among individual rats. In other words, perhaps the CTA hotspot for one rat is slightly more anterior or posterior in IC than another. Another possibility is that there is variability among the signals and/or strategies that individual rats use to “solve the task” and IC is only involved with processing certain types of information. We tried our best to convey these points in the Discussion section.

13. What is the rationale for the analysis in Figure 6? Data lack quantitative evaluation. Caption appears to be missing text?

Response: It is unfortunate the caption for Figure 7 (previously Figure 6) was cut off in the editorial process. The rationale for the analysis in Figure 7 (previously Figure 6) was to visualize behavioral patterns of all groups (left column; group-wise performance comparison) and individual animals in LiCl-sham group (middle column) and LiCl-IC2+IC3 group (right column) across the series of tests. We have replaced the bar graphs in left column with box and whisker plots as suggested (Reviewer 2, Results #2) and quantitative evaluations are not included as they were done and denoted in the previous figures (Figures 4-6; previously Figures 3-5). Since middle and right columns are showing individual animals’ performance, they are not quantitatively evaluated.

Minor

Introduction

1. The distinction between “sensory” and “visceral” is not intuitive, as gustatory input is sometimes referred to as visceral, and visceral input is generally referred to as sensory. Might be more useful to refer to the distinct biological sources of the inputs evoked by the US and CS.

Response: Yes, this is a good point. We now indicate that we are referring to the orosensory properties of the ingested substance as the CS, and the negative interoceptive state produced by LiCl as the US in the introduction (see lines 53-55). Moreover, in accordance with the literature, we’ve used alternative terms such as general visceral or interoceptive stimuli to refer the US throughout the manuscript.

2. The term “oromotor and somatic responses” to refer to TR behavior is unclear; do you intend it to include both oral and somatic motor responses?

Response: Yes, we did. As listed in Table 4-1 (previously Supplementary Table 1), animals’ TR responses are not limited to the oromotor reactions. They also include somatic movements such as paw lick, chin rub, forelimb flail and head shake. Thus, we intentionally used the term “oromotor and somatic responses” to include both.

3. What is meant by “online (emphasis) integration"; does it not simply refer to step 3 in Figure 1?

Response: The term “online” was used to emphasize the fact that CS and US were given simultaneously, as opposed to the cases in which step 3 can arise from CSs and USs that are experienced with long delays in between.

Methods

4. How much total lost time was present in the videos?

Response: This was relatively rare. Of the 25 (out of 46) rats that had {greater than or equal to}1 second missing from a video on the taste-visceral US pairing session, the median time missing was 4.64 seconds per video. All rats included for analysis in taste-visceral pairing session had at least 91.5 out of 120 total seconds scored. For the 21 (out of 45) rats that had time missing from the retention test videos, the median time missing was 2.53 seconds per video. All rats included for analysis in retention test had at least 67.57 out of 90 seconds scored.

Results

5. Overall, it would help to have the presentation of results guided by explicit questions, allowing for a better judgement of the appropriateness of the approach and statistical evaluation. In Figures 3-5, it is unclear to me how exactly the letters indicate significant differences between groups. It should be relatively simple to incorporate all the relevant information from Tables 2-6 into these figures, for example by using common brackets and asterisks (rendering the tables unnecessary).

Response: We like the reviewer’s suggestion and have included specific goals to the beginning of each Results subsection. Moreover, we agree that using letters to indicate statistical difference can confuse the readers as both reviewers pointed out (Reviewer 2, Results #1). We have now replaced them with brackets and asterisks in Figures 4C, 4D, 5 and 6 (previously Figures 3C, 3D, 4, and 5) and corresponding captions have been revised. However, we have decided to keep the tables as a detailed report of statistical evaluation.

REVIEWER 2

Title:

1. The authors should consider adding the species to the title.

Response: We agree and have added the species to the title.

Methods:

1. Why were different volumes of ibotenic acid used for the surgeries in Phase 1 (0.18 μl) and Phase 2 (0.1 μl)? Were there any significant differences in the size and breadth of the lesions between these two surgical groups?

Response: Phase 2 was conducted after Phase 1 and we adjusted the surgical coordinates and injection volumes for phase 2 based the lesion placements observed in Phase 1. We did not find any statistical differences in the sizes of lesions in animals with IC2+IC3 lesion from the two phases (unpaired two-tailed t-test (a=0.05); p=0.6837 for lesion size of IC2+IC3, p=0.8647 for lesion size of IC2), and p=0.6333 for lesion size of IC3.

2. Please revise the sentence explaining the robust Z-score standardization. “In order to examine patterns of behavior across the multiple behavioral assessments, each individual rat’s behavior performance was converted to a robust Z-score standardized against the median and deviation of the LiCl Sham group the main behavioral measurements.”

Response: We have changed the sentence as requested in Data Analyses section under Materials and Methods (see lines 331-332).

3. I appreciated the authors method of correcting for the portions of trials when data could not be collected due to obscured views. However, I believe a couple of points of clarification would help with communication. The author’s description says “The ingestive and aversive TR scores from the videos that had >1 second of no data were corrected for lost time by dividing the total scores by scorable time and then multiplying that by 30.” First, were the ingestive and aversive TR scores summed (i.e. total scores) then divided by the scorable time or was the total ingestive TR score divided by the scorable time and the total aversive TR score divided by the scorable time? Second, I hate to admit but it took me far too long to understand why the scores were then multiplied by 30. The authors may wish to clarify here that this corresponds to the length of time of a single infusion trial.

Response: Individual behavior categories of the ingestive and aversive TR responses were summed and corrected separately. The scores were divided by the scorable time and then multiplied by 30, which is the duration of a single infusion. Then, they were summed to get total ingestive and aversive TR scores. We agree that our description was insufficient and have revised the sentences in Behavioral Analyses section under Materials and Methods (see lines 227-232).

4. Why did the authors choose to sum each rats Ingestive TR scores and Aversive TR scores across the 4 taste-visceral time-points and the 3 retention time-points? Wouldn’t finding the mean score for each rat per session allow for direct comparisons in Ingestive TR scores and Aversive TR scores between the two sessions?

Response: Based on prior application of this paradigm in the literature (Spector et al, 1988), we expected that ingestive responses would be high and aversive responses low in the taste-visceral US pairing session during the early part of the session, before the effects of the LiCl set in, and then shift as the LiCl took effect. The timing of intraperitoneal (IP) injection relative to the start of sucrose infusions was different between the two session types, which makes it difficult to draw direct comparisons between them.

Results Section:

1. I appreciate that the author’s provided tables showing the statistical differences for each experimental figure because the graphs were very confusing. For example, Figure 3C and 3D list letters (a; b; a,b) above the bar graphs with the caption saying “Different letters above the bars indicate a statistical difference (p<0.05) between groups.” However, according to the text and Table 4, the only significant difference for both the Ingestive Score and the Aversion Score was for the comparison between the NaCl group (written as Na instead of NaCl on the table) and the Sham-Li group. This same nomenclature is used for Figures 3CD, 4AB, and 5AB. Using black bars and asterisks to indicate significant differences would likely be easier to follow.

Response: Yes, we agree that using letters to indicate statistical difference can confuse the readers as both reviewers pointed out (Reviewer 1, Minor #5). We have now replaced them with brackets and asterisks in Figures 4C, 4D, 5 and 6 (previously Figures 3C, 3D, 4, and 5) and corresponding captions have been revised.

2. Relatedly, Figures 3CD, 4AB, and 5AB show each rats score with a bar graph at the median. However, the Semi-IQR is only shown when these plots are recreated in Figure 6. Furthermore, I understand that the mean and SEM are heavily influenced by outliers, but plotting the data as the median {plus minus} SIQR masks the variability of the data. The authors should consider remaking each figure as a box and whisker plot and indicate the mean for each group (with a plus sign for example) as well.

Response: We agree and have replaced the bar graphs with box and whisker plots in left column in Figure 7 (previously Figure 6) to deliver additional information in each graph (mean value is indicated with ’+’ symbol) and corresponding caption has been edited. However, we decided to keep Figures 4C, 4D, 5 and 6 (previously Figures 3C, 3D, 4, and 5) as a bar graph because we believe the individual data points displayed in these figures sufficiently show the distribution and variability of our data.

3. I appreciate the authors for providing the data as supplemental material for the incomplete groups of rats with either IC2 or IC3 lesions. For the most part, the authors are careful to refer to this supplementary information as a general “for comparison” (e.g. at the end of Results sections, Extensive Lesions in IC2+IC3 disrupted taste-guided oromotor and somatic responses to sucrose following IP LiCl injections and Two-Bottle Avoidance Test). However, they also use this incomplete data set (which they say were not used for analyses “due to the limited sample sizes.”) to directly compare with results from the complete IC2+IC3 data set at the end of Results sections, Taste Reactivity Test and Individual Rats with IC2+IC3 lesions that exhibit impaired avoidance generally display impairments on the prior measures of taste aversion. These statements should be revised to remove any interpretation of the incomplete data set to mirror the more general “for comparison” statements. Of course, the authors are free to move these points to the Discussion section if they wish to discuss how the results of the incomplete data set shape their interpretations of the IC2+IC3 data set.

Response: Yes, we agree. We have moved those sentences from the Results section to the Sources of Variability section under the General Discussion (see lines 568-570 and lines 574-577).

4. Unfortunately, I was unable to completely evaluate Figure 6 because the caption was cut off. I’m sure I would be better able to grasp the details of the middle and right columns with the complete figure caption.

Response: It is unfortunate the caption for Figure 7 (previously Figure 6) was cut off in the editorial process. We hope you are able to view the full caption in this iteration and it provides sufficient information. While the left column shows the behavioral patterns of all groups that allows readers to compare group-wise performance across the series of experiments, the middle and right columns show the performance of individual animals in LiCl-sham group (middle column) and LiCl-IC2+IC3 group (right column).

5. What do the “r” and “p” stand for in Table 7?

Response: “r” stands for the Spearman’s correlation coefficient and “p” stands for the statistical significance in Spearman’s rank-order correlation. We have added the information in the caption under Table 7.

Reference

Spector AC, Breslin P, Grill HJ (1988) Taste reactivity as a dependent measure of the rapid formation of conditioned taste aversion: a tool for the neural analysis of taste-visceral associations. Behav Neurosci 102:942-952.

## References

[B1] Allen GV, Saper CB, Hurley KM, Cechetto DF (1991) Organization of visceral and limbic connections in the insular cortex of the rat. J Comp Neurol 311:1–16. 10.1002/cne.903110102 1719041

[B2] Bales MB, Schier LA, Blonde GD, Spector AC (2015) Extensive gustatory cortex lesions significantly impair taste sensitivity to KCl and quinine but not to sucrose in rats. PLoS One 10:e0143419. 10.1371/journal.pone.0143419 26599914 PMC4657922

[B3] Benjamini Y, Hochberg Y (1995) Controlling the false discovery rate - a practical and powerful approach to multiple testing. J R Stat Soc Series B Stat Methodol 57:289–300. 10.1111/j.2517-6161.1995.tb02031.x

[B4] Bermudez-Rattoni F, McGaugh JL (1991) Insular cortex and amygdala lesions differentially affect acquisition on inhibitory avoidance and conditioned taste aversion. Brain Res 549:165–170. 10.1016/0006-8993(91)90616-4 1654172

[B5] Boughter JD Jr, Fletcher M (2021) Rethinking the role of taste processing in insular cortex and forebrain circuits. Curr Opin Physiol 20:52–56. 10.1016/j.cophys.2020.12.009 33681544 PMC7932132

[B6] Braun JJ, Lasiter PS, Kiefer SW (1982) The gustatory neocortex of the rat. Physiol Psychol 10:13–45. 10.3758/BF03327004

[B7] Cubero I, Thiele TE, Bernstein IL (1999) Insular cortex lesions and taste aversion learning: effects of conditioning method and timing of lesion. Brain Res 839:323–330. 10.1016/s0006-8993(99)01745-x 10519056

[B8] Dunn LT, Everitt BJ (1988) Double dissociations of the effects of amygdala and insular cortex lesions on conditioned taste aversion, passive avoidance, and neophobia in the rat using the excitotoxin ibotenic acid. Behav Neurosci 102:3–23. 10.1037/0735-7044.102.1.3 3281693

[B9] Fontanini A, Katz DB (2006) State-dependent modulation of time-varying gustatory responses. J Neurophysiol 96:3183–3193. 10.1152/jn.00804.2006 16928791

[B10] Fontanini A, Katz DB (2009) Behavioral modulation of gustatory cortical activity. Ann N Y Acad Sci 1170:403–406. 10.1111/j.1749-6632.2009.03922.x 19686167 PMC2845300

[B11] Fresquet N, Angst MJ, Sandner G (2004) Insular cortex lesions alter conditioned taste avoidance in rats differentially when using two methods of sucrose delivery. Behav Brain Res 153:357–365. 10.1016/j.bbr.2003.12.011 15265630

[B12] Garcia J, Kimeldorf DJ, Koelling RA (1955) Conditioned aversion to saccharin resulting from exposure to gamma radiation. Science 122:157–158. 14396377

[B13] Geddes RI, Han L, Baldwin AE, Norgren R, Grigson PS (2008) Gustatory insular cortex lesions disrupt drug-induced, but not lithium chloride-induced, suppression of conditioned stimulus intake. Behav Neurosci 122:1038–1050. 10.1037/a0012748 18823161 PMC3684281

[B14] Gehrlach DA, Weiand C, Gaitanos TN, Cho E, Klein AS, Hennrich AA, Conzelmann KK, Gogolla N (2020) A whole-brain connectivity map of mouse insular cortex. Elife 9:e55585. 10.7554/eLife.55585 32940600 PMC7538160

[B15] Gogolla N (2017) The insular cortex. Curr Biol 27:R580–R586. 10.1016/j.cub.2017.05.010 28633023

[B16] Grigson PS, Reilly S, Shimura T, Norgren R (1998) Ibotenic acid lesions of the parabrachial nucleus and conditioned taste aversion: further evidence for an associative deficit in rats. Behav Neurosci 112:160–171. 9517824

[B17] Grill HJ, Norgren R (1978) The taste reactivity test. I. Mimetic responses to gustatory stimuli in neurologically normal rats. Brain Res 143:263–279. 10.1016/0006-8993(78)90568-1 630409

[B18] King CT, Hashimoto K, Blonde GD, Spector AC (2015) Unconditioned oromotor taste reactivity elicited by sucrose and quinine is unaffected by extensive bilateral damage to the gustatory zone of the insular cortex in rats. Brain Res 1599:9–19. 10.1016/j.brainres.2014.12.035 25536305 PMC4344848

[B19] Krushel LA, van der Kooy D (1988) Visceral cortex: integration of the mucosal senses with limbic information in the rat agranular insular cortex. J Comp Neurol 270:39–54. 10.1002/cne.902700105 2453537

[B20] Lasiter PS, Glanzman DL (1982) Cortical substrates of taste aversion learning: dorsal prepiriform (insular) lesions disrupt taste aversion learning. J Comp Physiol Psychol 96:376–392. 10.1037/h0077894 6284812

[B21] Livneh Y, Sugden AU, Madara JC, Essner RA, Flores VI, Sugden LA, Resch JM, Lowell BB, Andermann ML (2020) Estimation of current and future physiological states in insular cortex. Neuron 105:1094–1111.e10. 10.1016/j.neuron.2019.12.027 31955944 PMC7083695

[B22] Naor C, Dudai Y (1996) Transient impairment of cholinergic function in the rat insular cortex disrupts the encoding of taste in conditioned taste aversion. Behav Brain Res 79:61–67. 10.1016/0166-4328(95)00262-6 8883817

[B23] Nerad L, Ramírez-Amaya V, Ormsby CE, Bermúdez-Rattoni F (1996) Differential effects of anterior and posterior insular cortex lesions on the acquisition of conditioned taste aversion and spatial learning. Neurobiol Learn Mem 66:44–50. 10.1006/nlme.1996.0042 8661250

[B24] Parker LA (2003) Taste avoidance and taste aversion: evidence for two different processes. Learn Behav 31:165–172. 10.3758/bf03195979 12882375

[B25] Parkes SL, Bradfield LA, Balleine BW (2015) Interaction of insular cortex and ventral striatum mediates the effect of incentive memory on choice between goal-directed actions. J Neurosci 35:6464–6471. 10.1523/JNEUROSCI.4153-14.2015 25904797 PMC4405558

[B200] Paxinos G, Watson C (2007) The Rat Brain in Stereotaxic Coordinates. 6th ed. San Diego: Academic.

[B26] Pelchat ML, Grill HJ, Rozin P, Jacobs J (1983) Quality of acquired responses to tastes by Rattus norvegicus depends on type of associated discomfort. J Comp Psychol 97:140–153. 6307586

[B27] Phillips MI, Norgren RE (1970) A rapid method for permanent implantation of an intraoral fistula in rats. Behav Res Meth Instru 2:124. 10.3758/BF03211020

[B28] Reilly S, Bornovalova MA (2005) Conditioned taste aversion and amygdala lesions in the rat: a critical review. Neurosci Biobehav Rev 29:1067–1088. 10.1016/j.neubiorev.2005.03.025 15893375

[B29] Reilly S, Grigson PS, Norgren R (1993) Parabrachial nucleus lesions and conditioned taste aversion: evidence supporting an associative deficit. Behav Neurosci 107:1005–1017. 10.1037/0735-7044.107.6.1005 8136054

[B30] Roman C, Reilly S (2007) Effects of insular cortex lesions on conditioned taste aversion and latent inhibition in the rat. Eur J Neurosci 26:2627–2632. 10.1111/j.1460-9568.2007.05872.x 17970726

[B31] Rosenblum K, Schul R, Meiri N, Hadari YR, Zick Y, Dudai Y (1995) Modulation of protein tyrosine phosphorylation in rat insular cortex after conditioned taste aversion training. Proc Natl Acad Sci U S A 92:1157–1161. 10.1073/pnas.92.4.1157 7862652 PMC42657

[B32] Rosenblum K, Berman DE, Hazvi S, Lamprecht R, Dudai Y (1997) NMDA receptor and the tyrosine phosphorylation of its 2B subunit in taste learning in the rat insular cortex. J Neurosci 17:5129–5135. 9185550 10.1523/JNEUROSCI.17-13-05129.1997 PMC6573317

[B33] Schafe GE, Bernstein IL (1998) Forebrain contribution to the induction of a brainstem correlate of conditioned taste aversion. II. Insular (gustatory) cortex. Brain Res 800:40–47. 10.1016/S0006-8993(98)00492-2 9685579

[B34] Schier LA, Spector AC (2019) The functional and neurobiological properties of bad taste. Physiol Rev 99:605–663. 10.1152/physrev.00044.2017 30475657 PMC6442928

[B35] Schier LA, Hashimoto K, Bales MB, Blonde GD, Spector AC (2014) High-resolution lesion-mapping strategy links a hot spot in rat insular cortex with impaired expression of taste aversion learning. Proc Natl Acad Sci U S A 111:1162–1167. 10.1073/pnas.1315624111 24395785 PMC3903191

[B36] Schier LA, Blonde GD, Spector AC (2016) Bilateral lesions in a specific subregion of posterior insular cortex impair conditioned taste aversion expression in rats. J Comp Neurol 524:54–73. 10.1002/cne.23822 26053891 PMC4659750

[B37] Schier LA, Hyde KM, Spector AC (2019) Conditioned taste aversion versus avoidance: a re-examination of the separate processes hypothesis. PLoS One 14:e0217458. 10.1371/journal.pone.0217458 31216290 PMC6583984

[B38] Spector AC, Breslin P, Grill HJ (1988) Taste reactivity as a dependent measure of the rapid formation of conditioned taste aversion: a tool for the neural analysis of taste-visceral associations. Behav Neurosci 102:942–952. 10.1037/0735-7044.102.6.942 2850815

[B39] Spector AC, Norgren R, Grill HJ (1992) Parabrachial gustatory lesions impair taste aversion learning in rats. Behav Neurosci 106:147–161. 10.1037/0735-7044.106.1.147 1313242

[B40] Vincis R, Fontanini A (2016) Associative learning changes cross-modal representations in the gustatory cortex. Elife 5:e16420. 10.7554/eLife.16420 27572258 PMC5026467

[B41] Vincis R, Chen K, Czarnecki L, Chen J, Fontanini A (2020) Dynamic representation of taste-related decisions in the gustatory insular cortex of mice. Curr Biol 30:1834–1844.e5. 10.1016/j.cub.2020.03.012 32243860 PMC7239762

[B42] Yamamoto T, Matsuo R, Kawamura Y (1980) Localization of cortical gustatory area in rats and its role in taste discrimination. J Neurophysiol 44:440–455. 10.1152/jn.1980.44.3.440 7441309

