# Supplementary material for: A Subregion of Insular Cortex Is Required for Rapid Taste-Visceral Integration and Consequent Conditioned Taste Aversion and Avoidance Expression in Rats
Source: eNeuro. 2022 Jul 6;9(4):ENEURO.0527-21.2022. doi: 10.1523/ENEURO.0527-21.2022 (PMC9267001; doi:10.1523/ENEURO.0527-21.2022)
Supplement: Extended Data Figure 4-4 — Definitions of oromotor and somatic reactivity behaviors. Corresponds to Figures 4 and 5. Download Figure 4-4, DOC file. [file enu-eN-NWR-0527-21-s02.doc]

| Behavior | Description |  |
| --- | --- | --- |
| *Ingestive* |  |  |
| Tongue protrusion (tp) | Rhythmic protrusions of tongue on the midline. | |
| Lateral tongue protrusion (ltp) | Non-rhythmic extensions of tongue that are morphologically distinct from tongue protrusions. The tongue appears on either side of the mouth resulting in an asymmetrical separation of the lips. | |
| Mouth movement (mm) | Rhythmic openings of the mandible with low amplitude, often visible via the movement of jaw. | |
| Paw lick (pl) | Rhythmic, large amplitude extensions of tongue on the midline directed at the forepaws (approximately 6 times/s), though occasionally this action is directed at the floor or other substrate. Paw licking is distinguished from grooming of the face by the fact that the tongue moves to the forepaws. Paw licking is scored by duration (in second), which is then multiplied by 6. | |
| *Aversive* |  |  |
| Gape (g) | Large amplitude openings of the mandible revealing upper and lower incisors with concomitant retraction of the lateral corners of the mouth. | |
| Chin rub (cr) | The mouth is brought to a direct contact with the floor or wall of the chamber and the body is projected forward by the flexion of the dorsal neck and forelimb musculature. Fluid is often deposited during this action as animals rub their face/mouth on the floor. | |
| Forelimb flail (ff) | A burst of high frequency (>60 Hz) movements of one or both forelimbs in a left-right motion. | |
| Head shake (hs) | A burst of high frequency (>60 Hz) side-to-side movements of the head. Resembles a lateral/horizontal rotation of the head and neck. | |
| *Non-ingestive* |  |  |
| Passive drip (pd) | The accumulation of fluid at the tip of the lower mandible that drips onto the bottom of the chamber in the absence of any other overt behavior. This response is scored by duration (in s). | |
| No data (nd) | The absence of an animal for at least one second in the video. This is caused by the animal's movement, positioning, or obstruction in video due to a substance in the filmed frames. | |

Figure 4-4. Definitions of Oromotor and Somatic Reactivity Behaviors

*Notes*. Corresponds to Figures 4 and 5.
