# Supplementary figures and images for: A Subregion of Insular Cortex Is Required for Rapid Taste-Visceral Integration and Consequent Conditioned Taste Aversion and Avoidance Expression in Rats
Source: eNeuro. 2022 Jul 6;9(4):ENEURO.0527-21.2022. doi: 10.1523/ENEURO.0527-21.2022 (PMC9267001; doi:10.1523/ENEURO.0527-21.2022)

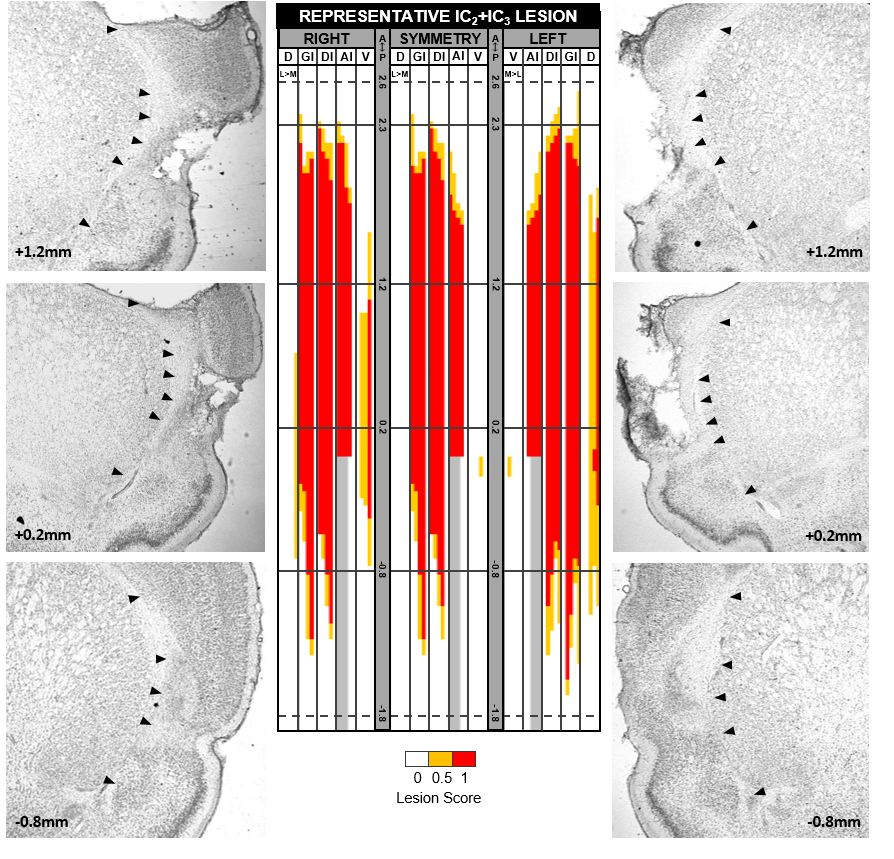

Supplement: Extended Data Figure 3-1 — Representative IC2+IC3 lesion map and corresponding photomicrographs of brain sections in both hemispheres. IC2+IC3 lesion brain sections at the coordinates of +1.2 mm (top), +0.2 mm (middle), –0.8 mm (bottom) in the left and right hemispheres (right and left column, respectively) with black arrowheads indicating the borders of each layer in IC and surrounding region. Middle column shows representative IC2+IC3 lesion maps of each hemisphere with a symmetry lesion map in the middle. Solid and dotted lines on the map indicate different AP levels relative to bregma including IC2 borders (+1.2 and +0.2 mm) and IC3 borders (+0.2 and –0.8 mm). A, anterior to bregma, P, posterior to bregma, D, dorsal to the granular layer (GI); GI, granular IC; DI, dysgranular IC; AI, agranular IC (dorsal to the rhinal fissure); V, ventral to rhinal fissure. Download Figure 3-1, TIF file. [file enu-eN-NWR-0527-21-s07.tif]

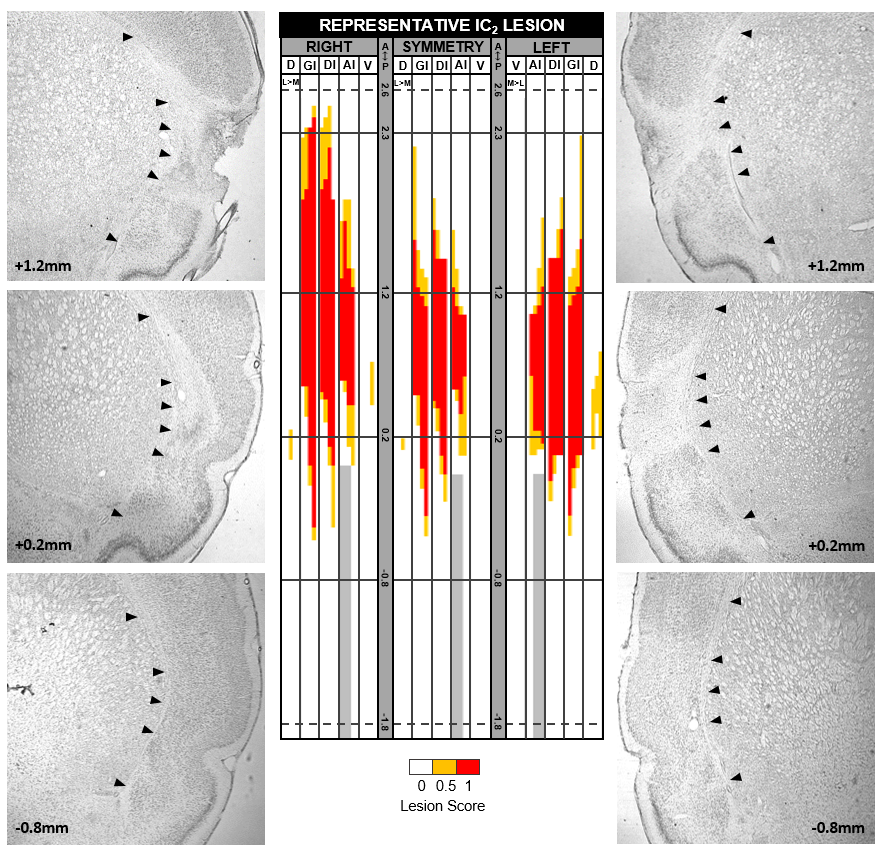

Supplement: Extended Data Figure 3-2 — Representative IC2 lesion map and corresponding photomicrographs of brain sections in both hemispheres. IC2 lesion brain sections at the coordinates of +1.2 mm (top), +0.2 mm (middle), –0.8 mm (bottom) in the left and right hemispheres (right and left column, respectively) with black arrowheads indicating the borders of each layer in IC and surrounding region. Middle column shows representative IC2 lesion maps of each hemisphere with a symmetry lesion map in the middle. Solid and dotted lines on the map indicate different AP levels relative to bregma including IC2 borders (+1.2 and +0.2 mm) and IC3 borders (+0.2 and –0.8 mm). A, anterior to bregma, P, posterior to bregma, D, dorsal to the granular layer (GI); GI, granular IC; DI, dysgranular IC; AI, agranular IC (dorsal to the rhinal fissure); V, ventral to rhinal fissure. Download Figure 3-2, TIF file. [file enu-eN-NWR-0527-21-s08.tif]

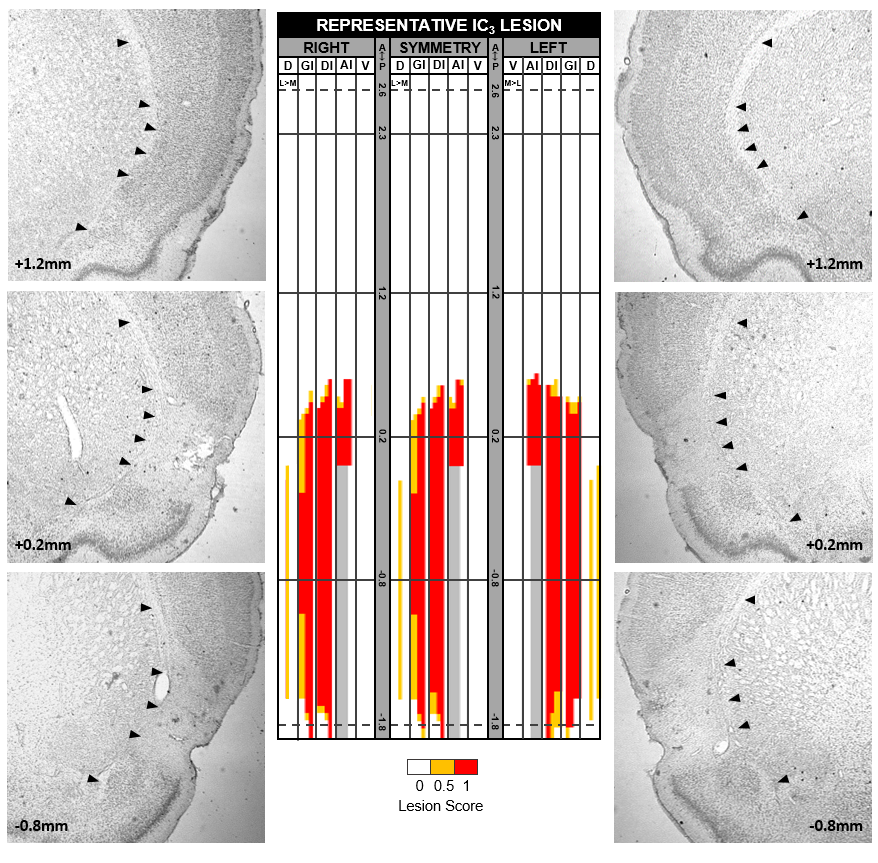

Supplement: Extended Data Figure 3-3 — Representative IC3 lesion map and corresponding photomicrographs of brain sections in both hemispheres. IC3 lesion brain sections at the coordinates of +1.2 mm (top), +0.2 mm (middle), –0.8 mm (bottom) in the left and right hemispheres (right and left column, respectively) with black arrowheads indicating the borders of each layer in IC and surrounding region. Middle column shows representative IC3 lesion maps of each hemisphere with a symmetry lesion map in the middle. Solid and dotted lines on the map indicate different AP levels relative to bregma including IC2 borders (+1.2 and +0.2 mm) and IC3 borders (+0.2 and –0.8 mm). A, anterior to bregma, P, posterior to bregma, D, dorsal to the granular layer (GI); GI, granular IC; DI, dysgranular IC; AI, agranular IC (dorsal to the rhinal fissure); V, ventral to rhinal fissure. Download Figure 3-3, TIF file. [file enu-eN-NWR-0527-21-s09.tif]

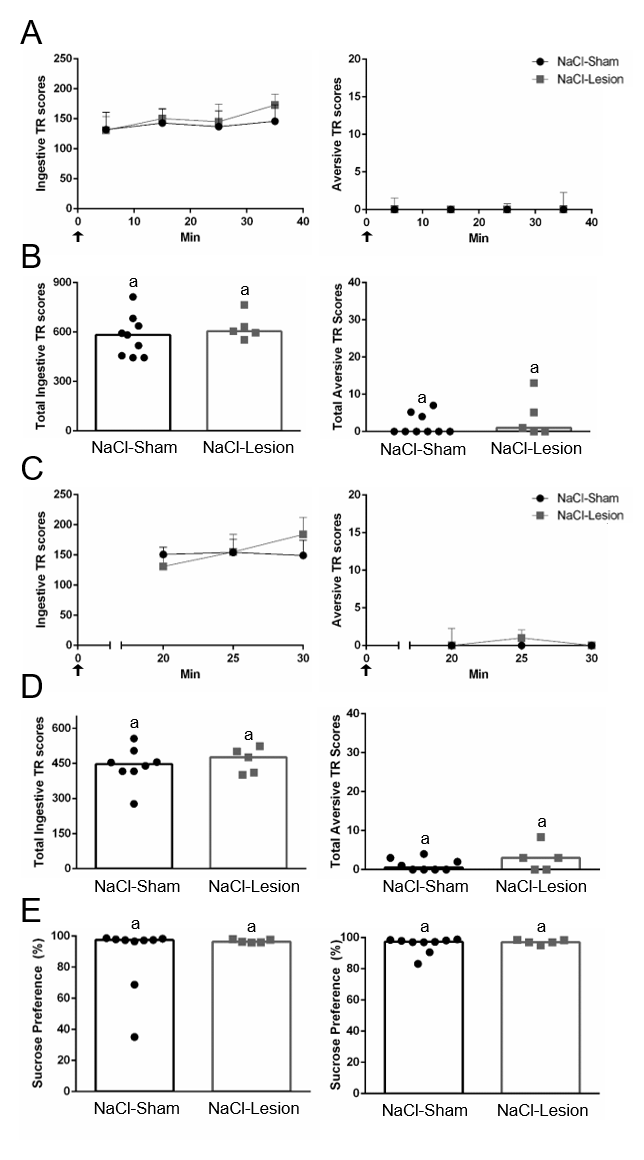

Supplement: Extended Data Figure 4-1 — Lesions in IC2 and/or IC3 did not affect the behavior performance in NaCl-injected rats. A, Median (+ Semi-IQR) ingestive (left) and aversive (right) TR scores to IO sucrose infusions in acquisition session following NaCl injection (black arrow) in sham (n = 9) or lesion group (n = 5). B, Median total ingestive (left) and aversive (right) TR scores during acquisition are plotted with the data points indicating individual animals. C, Median (+ Semi-IQR) ingestive (left) and aversive (right) TR scores in retention session as a function of time following the LiCl injection (black arrow) in sham (n = 8) or lesion group (n = 5). D, Median total ingestive (left) and aversive (right) TR scores during retention are plotted with individual data points. E, Median sucrose preference over water (in percentage) during the first 24 h (left) or 48 h (right) in two-bottle test are plotted with individual data points. A–E, Histograms with the same letter were not statistically different (all ps > 0.05). Statistics are in Extended Data Figure 4-5. Download Figure 4-1, TIF file. [file enu-eN-NWR-0527-21-s10.tif]

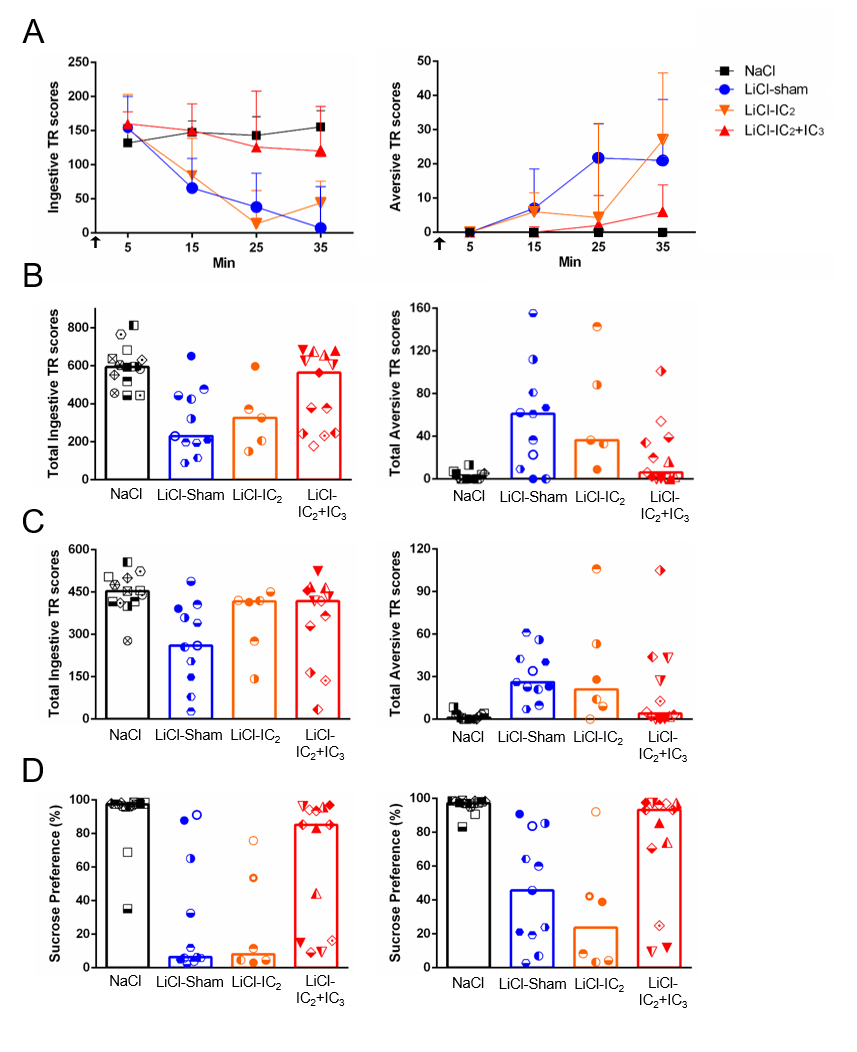

Supplement: Extended Data Figure 4-2 — A, Median (+ Semi-IQR) ingestive (left) and aversive (right) TR scores across the taste-visceral pairing session following the intraperitoneal injection of either NaCI or LiCI (black arrow) are plotted. B, C, Median value of total ingestive (left) and aversive (right) TR scores during pairing (B) and retention (C) are plotted with different symbols for individual animals in each group. D, Median sucrose preferences over water in percentage during the first 24 h (left) or 48 h (right) of two-bottle test are plotted with individual data points. NaCl group (n = 13–14), LiCl-sham group (n = 11), LiCl-IC2 group (n = 5–6), and LiCl- IC2+IC3 group (n = 12–13). Download Figure 4-2, TIF file. [file enu-eN-NWR-0527-21-s11.tif]

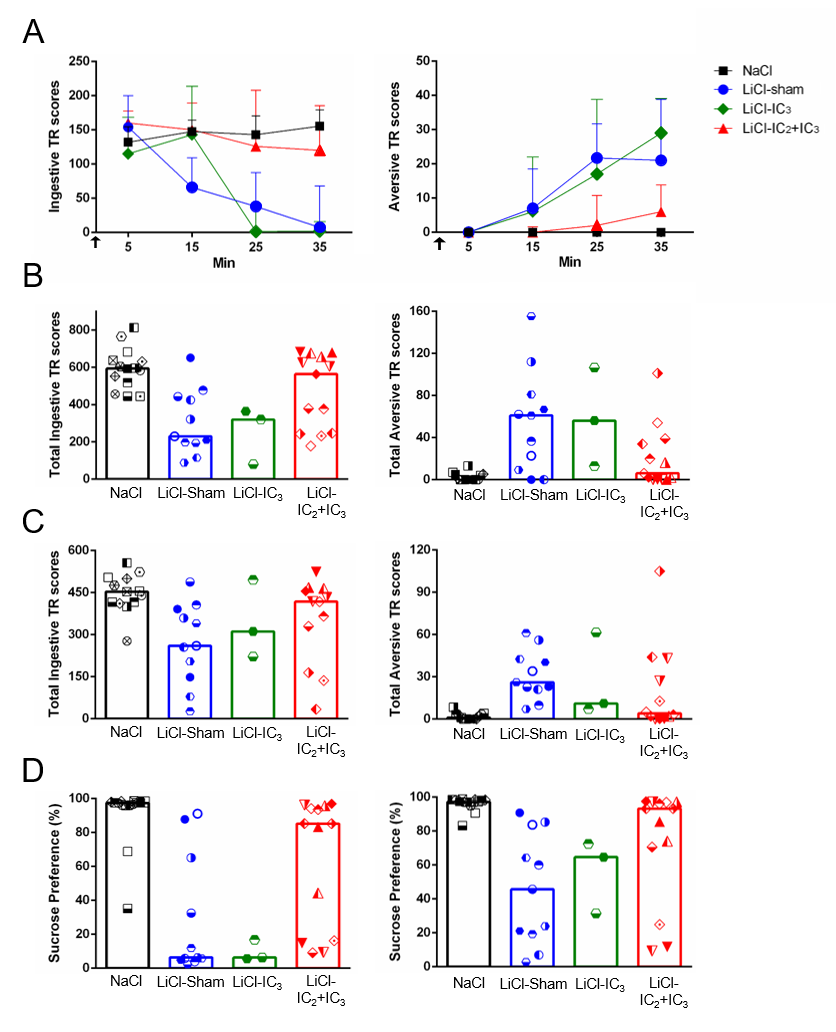

Supplement: Extended Data Figure 4-3 — A, Median (+ Semi-IQR) ingestive (left) and aversive (right) TR scores across the taste-visceral pairing session following the intraperitoneal injection of either NaCI or LiCI (black arrow) are plotted. B, C, Median value of total ingestive (left) and aversive (right) TR scores during pairing (B) and retention (C) are plotted with different symbols for individual animals in each group. D, Median sucrose preferences over water in percentage during the first 24 h (left) or 48 h (right) of two-bottle test are plotted with individual datapoints. NaCl group (n = 13–14), LiCl-sham group (n = 11), LiCl-IC3 group (n = 3), and LiCl- IC2+IC3 group (n = 12–13). Download Figure 4-3, TIF file. [file enu-eN-NWR-0527-21-s12.tif]

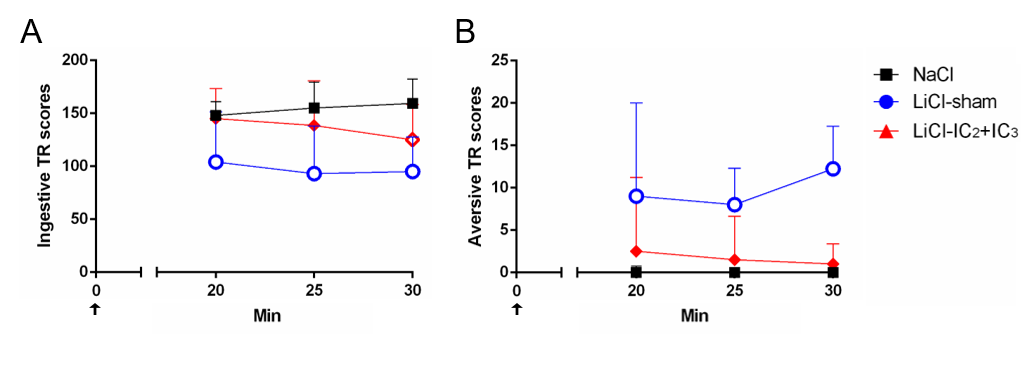

Supplement: Extended Data Figure 5-1 — A, B, Median (+ Semi-IQR) ingestive and aversive TR scores to IO sucrose infusions in retention session are plotted as a function of time following the intraperitoneal injection of either NaCI (n = 13) or LiCI (n = 11 for sham, n = 12 for IC2+IC3 lesion; black arrow). Open symbol is assigned for LiCl-injected groups at each timepoint if there is statistical significance (p ≤ 0.05) compared to the NaCl group. Statistics are in Extended Data Figures 5-2 and 5-3. Download Figure 5-1, TIF file. [file enu-eN-NWR-0527-21-s13.tif]

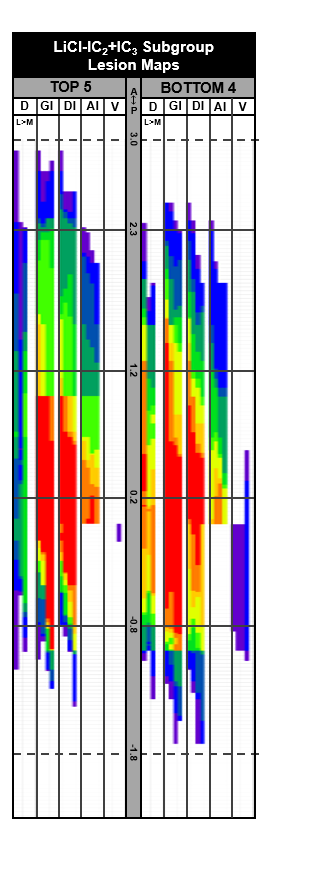

Supplement: Extended Data Figure 6-2 — Comparison of lesion sites between two subgroups of LiCl-injected animals with IC2 and IC3 lesions. Group-wise overlap lesion maps showing the average lesion scores in color-coded manner on 2D lesion mapping grids are presented for the rats in LiCl-IC2+IC3 group with the greatest impairment (“Top 5”; individual symbols: ◇◭⧩◆◮) and those with the least impairment (“Bottom 4”; individual symbols: ⬗⬘⬙⟐) in the first 24 h of the two-bottle choice test. Solid and dotted lines indicate different AP levels relative to bregma including IC2 borders (+1.2 and +0.2 mm) and IC3 borders (+0.2 and –0.8 mm). A, anterior to bregma, P, posterior to bregma, D, dorsal to the granular layer (GI); GI, granular IC; DI, dysgranular IC; AI, agranular IC (dorsal to the rhinal fissure); V, ventral to rhinal fissure. Download Figure 6-2, TIF file. [file enu-eN-NWR-0527-21-s15.tif]

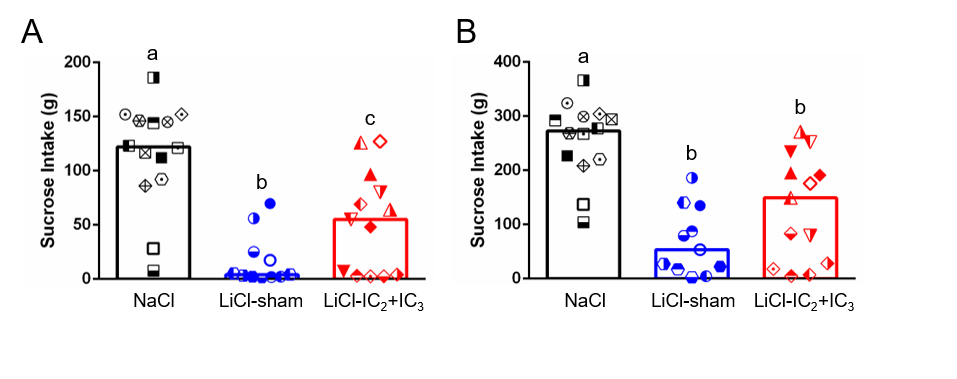

Supplement: Extended Data Figure 6-1 — Animals with IC2 and IC3 lesions given LiCl at the initial pairing failed to avoid sucrose consumption on the two-bottle choice retention test. Median values of sucrose intake (g) during the first 24 h (A) or 48 h (B) in two-bottle test are plotted with different symbols for individual animals in NaCl group (n = 14), LiCl-sham control group (n = 11) and LiCl-IC2+IC3 lesion group (n = 13). Different letters above the bars indicate statistical difference (p ≤ 0.05) between groups. Statistics are on Extended Data Figure 6-3. Download Figure 6-1, TIF file. [file enu-eN-NWR-0527-21-s14.tif]

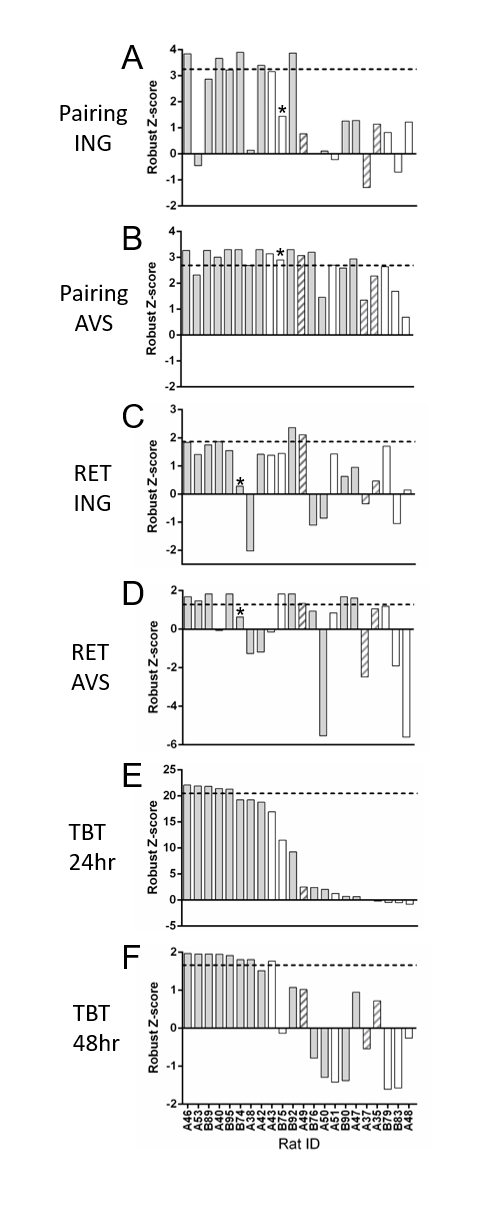

Supplement: Extended Data Figure 7-1 — Standardized behavior scores of LiCl-given animals with IC2 and/or IC3 lesion. The robust Z-scores of LiCl-injected lesion animals on ingestive (A) and aversive TR (B) in the stimulus pairing session, ingestive TR (C) and aversive TR (D) in retention and sucrose preference over water during the first 24 hour (E) and 48 hour (F) in two bottle test are plotted. For aversive TRs, (B) and (D), the signs of Z scores are reversed so that the positive values indicate more impairment in their performance. The animals are arranged in order of showing high to low sucrose preference in 24-hour two bottle test and their IDs are denoted on the x axis in (F). Animals in phase 1 start with ’A’ and animals in phase 2 start with ’B’. Bar appearance is different for each lesion group (IC2: open, IC3: hatched, IC2+IC3: filled with gray color). Asterisk above the bar indicates incomplete data. Abbreviations: RET, retention; TBT, two bottle test; ING, ingestive TR; AVS, aversive TR. Download Figure 7-1, TIF file. [file enu-eN-NWR-0527-21-s16.tif]
