# Supplementary material for: A Subregion of Insular Cortex Is Required for Rapid Taste-Visceral Integration and Consequent Conditioned Taste Aversion and Avoidance Expression in Rats
Source: eNeuro. 2022 Jul 6;9(4):ENEURO.0527-21.2022. doi: 10.1523/ENEURO.0527-21.2022 (PMC9267001; doi:10.1523/ENEURO.0527-21.2022)
Supplement: Extended Data Figure 5-2 — Comparison of ingestive TR scores between groups at select time points across the retention test. Corresponds to Figure 5. Download Figure 5-2, DOC file. [file enu-eN-NWR-0527-21-s04.doc]

Extended Figure 5-2. Comparison of Ingestive TR Scores between Groups at Select Time points across the Retention Test

| Minute 25, q* = 0.0167 | | | |
| --- | --- | --- | --- |
|  | Na | Sham-Li | IC_2_+IC_3_-Li |
| Na |  | 0.0014+ | 0.1260 |
| Sham-Li |  |  | 0.1872 |
| IC_2_+IC_3_-Li |  |  |  |

| Minute 30, q* = 0.0333 | | | |
| --- | --- | --- | --- |
|  | Na | Sham-Li | IC_2_+IC_3_-Li |
| Na |  | 0.0009+ | 0.0092+ |
| Sham-Li |  |  | 0.4038 |
| IC_2_+IC_3_-Li |  |  |  |

*Notes.* Corresponds to Extended Figure 5, and 5-1. Significance level was adjusted based on Benjamini-Hochberg false discovery rate for multiple comparisons (q*; Benjamini and Hochberg, 1995). Values with a plus symbol (+) are statistically significant after correction.

| Minute 20, q* = 0.0167 | | | |
| --- | --- | --- | --- |
|  | Na | Sham-Li | IC_2_+IC_3_-Li |
| Na |  | 0.0048+ | 0.7182 |
| Sham-Li |  |  | 0.1462 |
| IC_2_+IC_3_-Li |  |  |  |
