# Supplementary material for: A Subregion of Insular Cortex Is Required for Rapid Taste-Visceral Integration and Consequent Conditioned Taste Aversion and Avoidance Expression in Rats
Source: eNeuro. 2022 Jul 6;9(4):ENEURO.0527-21.2022. doi: 10.1523/ENEURO.0527-21.2022 (PMC9267001; doi:10.1523/ENEURO.0527-21.2022)
Supplement: Extended Data Figure 5-3 — Comparison of aversive TR scores between groups at select time points across the retention test. Corresponds to Figure 5 and Extended Data Figure 5-1. Download Figure 5-3, DOC file. [file enu-eN-NWR-0527-21-s05.doc]

Extended Figure 5-3. Comparison of Aversive TR Scores between Groups at Select Time points across the Retention Test

| Minute 20, q* = 0.0167 | | | |
| --- | --- | --- | --- |
|  | Na | Sham-Li | IC_2_+IC_3_-Li |
| Na |  | 0.0004+ | 0.0418 |
| Sham-Li |  |  | 0.1817 |
| IC_2_+IC_3_-Li |  |  |  |

| Minute 25, q* = 0.0167 | | | |
| --- | --- | --- | --- |
|  | Na | Sham-Li | IC_2_+IC_3_-Li |
| Na |  | <0.0001+ | 0.1236 |
| Sham-Li |  |  | 0.0591 |
| IC_2_+IC_3_-Li |  |  |  |

| Minute 30, q* = 0.05 | | | |
| --- | --- | --- | --- |
|  | Na | Sham-Li | IC_2_+IC_3_-Li |
| Na |  | <0.0001+ | 0.0373 |
| Sham-Li |  |  | 0.0424+ |
| IC_2_+IC_3_-Li |  |  |  |

*Notes.* Corresponds to Figures 5 and 5-1. Significance level was adjusted based on Benjamini-Hochberg false discovery rate for multiple comparisons (q*; Benjamini and Hochberg, 1995). Values with a plus symbol (+) are statistically significant after correction.
