# Supplementary material for: A Subregion of Insular Cortex Is Required for Rapid Taste-Visceral Integration and Consequent Conditioned Taste Aversion and Avoidance Expression in Rats
Source: eNeuro. 2022 Jul 6;9(4):ENEURO.0527-21.2022. doi: 10.1523/ENEURO.0527-21.2022 (PMC9267001; doi:10.1523/ENEURO.0527-21.2022)
Supplement: Extended Data Figure 6-3 — Comparison of sucrose intake between groups on the two-bottle choice test. Corresponds to Figure 6 and Extended Data Figure 6-1. Download Figure 6-3, DOC file. [file enu-eN-NWR-0527-21-s06.doc]

| First 24hr, q* = 0.05 | | | |
| --- | --- | --- | --- |
|  | Na | Sham-Li | IC_2_+IC_3_-Li |
| Na |  | <0.0001+ | 0.0022+ |
| Sham-Li |  |  | 0.0393+ |
| IC_2_+IC_3_-Li |  |  |  |

Extended Figure 6-3. Comparison of Sucrose Intake between Groups on the Two Bottle Choice Test

| Full 48hr, q* = 0.0333 | | | |
| --- | --- | --- | --- |
|  | Na | Sham-Li | IC_2_+IC_3_-Li |
| Na |  | <0.0001+ | 0.0008+ |
| Sham-Li |  |  | 0.0963 |
| IC_2_+IC_3_-Li |  |  |  |

*Notes.* Corresponds to Figure 6 and 6-1. Significance level was adjusted based on Benjamini-Hochberg false discovery rate for multiple comparisons (q*; Benjamini and Hochberg, 1995). Values with a plus symbol (+) are statistically significant after correction.
