# Supplementary material for: A Subregion of Insular Cortex Is Required for Rapid Taste-Visceral Integration and Consequent Conditioned Taste Aversion and Avoidance Expression in Rats
Source: eNeuro. 2022 Jul 6;9(4):ENEURO.0527-21.2022. doi: 10.1523/ENEURO.0527-21.2022 (PMC9267001; doi:10.1523/ENEURO.0527-21.2022)
Supplement: Extended Data Figure 4-5 — Comparison of the NaCl-injected groups on each behavioral test. Corresponds to Figures 4–6. Download Figure 4-5, DOC file. [file enu-eN-NWR-0527-21-s03.doc]

| Lesion-Na vs Sham-Na on the TR Retention and Two Bottle Test | | | | | | | | | | |
| --- | --- | --- | --- | --- | --- | --- | --- | --- | --- | --- |
|  | TR Retention | | | | | | | | TBT | |
|  | Ingestive | | | | Aversive | | | | 24hr | 48hr |
|  | Min 20 | Min 25 | Min 30 | Total | Min 20 | Min 25 | Min 30 | Total |
| p-value | 0.3372 | >0.9999 | 0.0932 | 0.9293 | 0.4452 | 0.2168 | 0.4872 | 0.4577 | 0.6533 | 0.8731 |

Figure 4-5. Comparison of the NaCl-injected Groups on each Behavioral Test

| Lesion-Na vs Sham-Na on the taste-visceral pairing session | | | | | | | | | | |
| --- | --- | --- | --- | --- | --- | --- | --- | --- | --- | --- |
|  | Ingestive | | | | | Aversive | | | | |
|  | Min 5 | Min 15 | Min 25 | Min 35 | Total | Min 5 | Min 15 | Min 25 | Min 35 | Total |
| p-value | 0.9710 | 0.4366 | 0.6943 | 0.1469 | 0.3636 | 0.7303 | 0.8352 | 0.1099 | 0.4006 | 0.4825 |

*Notes.* Corresponds to Figures 4 and 4-1.
